# Biofilm formation and antibiofilm strategies in the *Achromobacter* genus: a scoping review

**DOI:** 10.3389/fcimb.2026.1942991

**Published:** 2026-10-30

**Authors:** Vincent Jean-Pierre, Estelle Jumas-Bilak, Jean-Philippe Lavigne, Hélène Marchandin

**Affiliations:** 1 HydroSciences Montpellier, Univ Montpellier, CNRS, IRD, Service de Microbiologie et Hygiène Hospitalière, CHU Nîmes, Montpellier, France; 2 HydroSciences Montpellier, Univ Montpellier, CNRS, IRD, Service de Prévention des Infections et de la Résistance, CHU Montpellier, Montpellier, France; 3 VBIC, INSERM, Univ Montpellier, Service de Microbiologie et Hygiène Hospitalière, Plateforme MICRO&BIO, CHU Nîmes, Nîmes, France

**Keywords:** *Achromobacter*, antibiofilm strategies, biofilm, chronic colonization, polymicrobial interactions

## Abstract

Biofilm formation plays a major role in bacterial persistence across clinical and environmental settings, with particular significance in chronic infections. *Achromobacter* spp. are environment-borne emerging opportunistic pathogens increasingly recognized in biofilm-associated diseases. This scoping review summarizes evidence on the prevalence and determinants of biofilm formation in *Achromobacter* and examines the antibiofilm effects of various strategies. Following the Preferred Reporting Items for Systematic Reviews and Meta-Analyses extension for Scoping Reviews guidelines, a comprehensive search of the PubMed-MEDLINE database was conducted from inception to June 2026 using the terms “*Achromobacter*” AND “biofilm”. Of 124 records identified, 79 studies were included. Data interpretation was challenged by methodological heterogeneity and variable confidence in species identification. *In vitro*, *ex vivo*, and *in vivo* models have been used, yielding complementary but sometimes conflicting results, underscoring the need for standardized protocols. However, biofilm formation appears to be widespread in *Achromobacter*. Statistical analyses of results from comparable studies showed that biofilm-forming capacity is independent of the clinical (cystic fibrosis or not) origin of the isolates while *Achromobacter xylosoxidans*, the most commonly isolated species in human infections, displays a higher prevalence of biofilm-producing isolates than other species. Biofilm formation may vary over time during chronic colonization or environmental persistence, probably reflecting distinct selective pressures. Among them, polymicrobial environments are hotspots of biotic interactions, where *Achromobacter* sp. can affect the biofilm formation of other pathogens like *Pseudomonas aeruginosa*. Combating *Achromobacter* biofilm can rely on antibiotics, particularly carbapenems, trimethoprim-sulfamethoxazole, and cefiderocol while some non-antibiotic approaches also show promising experimental antibiofilm activity.

## 1 Introduction

*Achromobacter* spp. are obligately aerobic, non-fermenting Gram-negative bacilli belonging to the order *Burkholderiales* and the family *Alcaligenaceae* (Yabuuchi et al., 1974). The genus appears ubiquitous, with a wide distribution across natural environments, particularly in water and in soil where *Achromobacter* spp. can function as plant growth-promoting rhizobacteria (Bhat et al., 2022) and contribute to the bioremediation of contaminated soils (Atuchin et al., 2023) but also in anthropized, either domestic or hospital, environments (Amoureux et al., 2013; Abdouchakour et al., 2015; Armando et al., 2023). Since the first description of the genus in the early 1970s, its taxonomic diversity has expanded to 22 recognized species which remain challenging to identify accurately using routine diagnostic procedures (Sorlin et al., 2024). Among them, *Achromobacter xylosoxidans* is by far the species most commonly recovered from clinical samples worldwide (Amoureux et al., 2016b, 2016a; Nielsen et al., 2016; Sorlin et al., 2024; Bayless et al., 2025). This opportunistic pathogen is increasingly recognized in cystic fibrosis (CF) (Menetrey et al., 2021; Bonis and Hunter, 2022), where it contributes to chronic respiratory colonization, pulmonary exacerbations, heightened airway inflammation, and progressive lung function decline, with clinical outcomes comparable to those observed in patients colonized by *Pseudomonas aeruginosa* (Hansen et al., 2010; Lambiase et al., 2011; Firmida et al., 2016, 2017; Kerem et al., 2023). Beyond CF, *Achromobacter* causes a wide spectrum of opportunistic infections, particularly catheter-related bloodstream infections, pneumonia, wound infections, and ear, nose, and throat infections, especially in immunocompromised or hospitalized individuals (Amoureux et al., 2016b; Isler et al., 2020; Montoya-Hinojosa et al., 2024; Bayless et al., 2025; Bianco et al., 2026). Collectively, these clinical manifestations are widely recognized as biofilm-associated diseases (Høiby, 2017; Vestby et al., 2020), in which microbial persistence is driven by the formation of structured communities embedded in an extracellular polymeric matrix consisting mainly of polysaccharides, proteins, extracellular DNA, and lipids, either attached to surfaces or as free-floating aggregates (Flemming and Wingender, 2010; Nielsen et al., 2016, 2019). Within these biofilms, bacteria exhibit reduced metabolic activity and are protected by the matrix, which limits antibiotic penetration, immune clearance, and enhances survival under environmental stressors (Nielsen et al., 2016; Mataracı-Kara et al., 2025; Wackernagel et al., 2025). The clinical relevance of *Achromobacter* is further underscored by its intrinsic and acquired multidrug resistance, which, combined with within-sample antimicrobial resistance heterogeneity, also contributes to compromise the efficacy of many conventional antibiotics (Bayless et al., 2025; Dupont et al., 2015, 2017). In clinical settings, *Achromobacter* biofilm often occurs in polymicrobial environments, interacting with other bacterial species - most notably *P. aeruginosa* - as well as with other microbial communities. These interactions may modulate biofilm structure, antibiotic tolerance, and disease progression (Azevedo et al., 2014; Menetrey et al., 2020; Vandeplassche et al., 2020; Bellavita et al., 2023). The combination of polymicrobial interactions, intrinsic multidrug resistance, and biofilm-associated tolerance significantly reduces the efficacy of conventional therapies, even when planktonic cells appear susceptible, thereby representing a key persistence trait providing *Achromobacter* with a survival advantage in human infections, in both CF (Montoya-Hinojosa et al., 2024) and non-CF settings (Wackernagel et al., 2025).

In this scoping review, considering the overall growing interest in the topic (**Figure 1A**), we aim to provide a comprehensive summary of the current knowledge on *Achromobacter* biofilm formation across strain origins (CF, non-CF, and environmental), species, polymicrobial contexts, and available antibiotic and non-antibiotic antibiofilm approaches including statistical analyses of data from comparable studies, when applicable.

**Figure 1:**
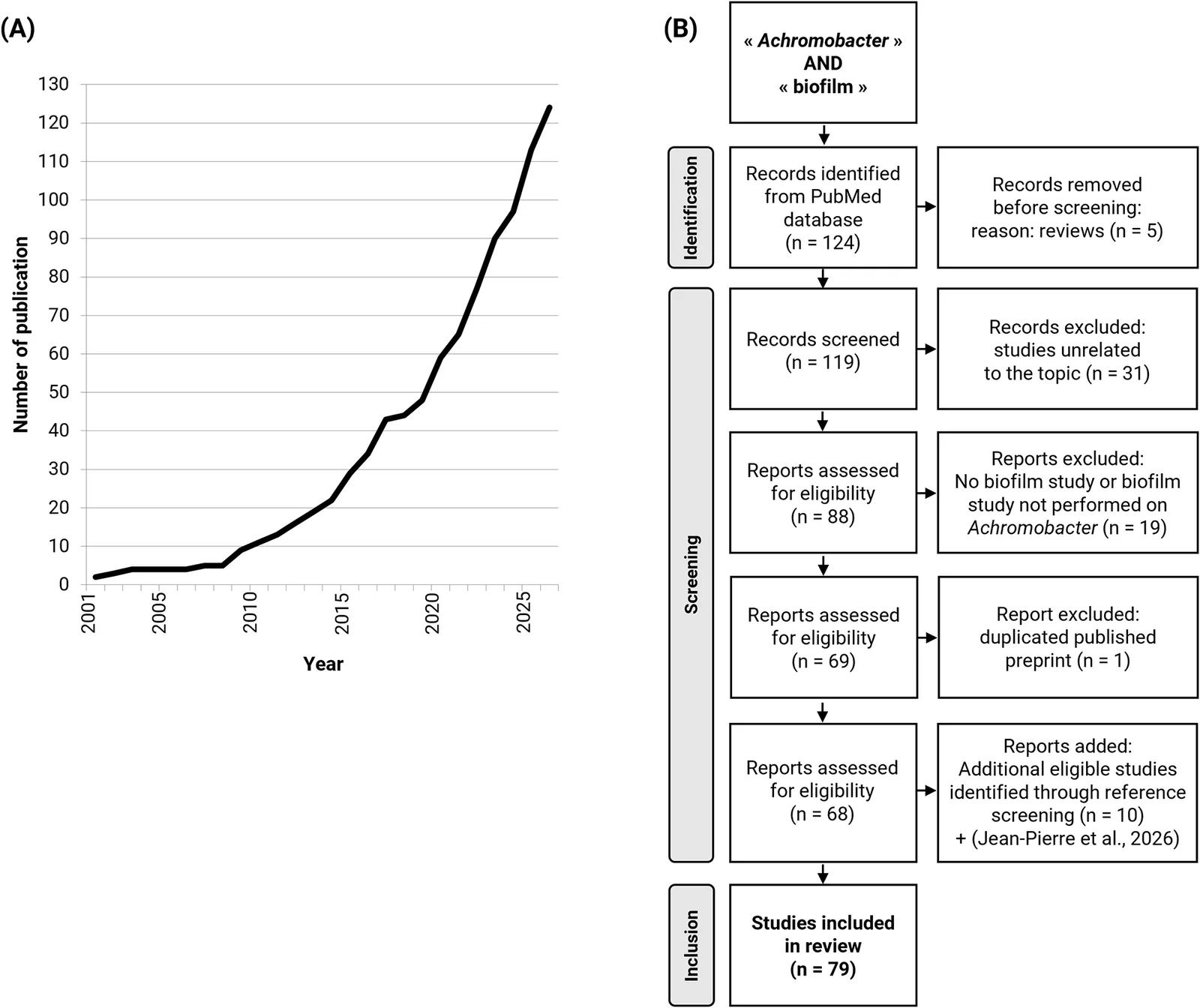
Results of the scoping review of the literature on “*Achromobacter*” AND “biofilm”. **(A)** Annual number of publications on *Achromobacter* biofilms among the 124 articles identified between January 2001 and June 2026, illustrating the growing interest in the field, particularly over the last five years. (**B)** Flow diagram of study selection (PubMed database; last search: June 14, 2026). n, number of publications.

## 2 Delineation of information gathered from the literature

This scoping review was performed in accordance with the Preferred Reporting Items for Systematic reviews and Meta-Analyses extension for Scoping Reviews (PRISMA-ScR) (Tricco et al., 2018).

### 2.1 Search strategy and study selection

A comprehensive search of the PubMed-Medline database was conducted using the terms “*Achromobacter*” AND “biofilm” to identify relevant studies published up to June 14, 2026. Titles and abstracts were independently screened by two reviewers (VJ-P and HM), to evaluate full-text eligibility. Any disagreements were resolved through discussion. Reference lists of the included articles were also manually screened to identify additional relevant studies.

Articles were included after full-text review if they met the following criteria: *i)* original research articles; *ii) in vitro*, *ex vivo* or *in vivo*/clinical studies investigating the ability of *Achromobacter* spp. to form biofilm, either alone or in interaction with other microorganisms; and/or *iii)* studies providing molecular insights into biofilm formation and regulation in *Achromobacter*; and/or *iv)* studies investigating antibiotic or non-antibiotic antibiofilm strategies targeting *Achromobacter*. Articles that did not meet these criteria were excluded.

### 2.2 Data extraction

Data were extracted using a predesigned spreadsheet corresponding to **Supplementary Table 1**. For each included study, the following information was collected, where applicable: publication year, authors, number of *Achromobacter* isolates, species identification, identification methods with specification of their accuracy for species identification in the *Achromobacter* genus, isolate sources, biofilm assessment methods, key findings regarding biofilm formation, polymicrobial biofilm interactions, and antibiotic and non-antibiotic antibiofilm strategies.

### 2.3 Overview and characteristics of the included studies

A total of 124 records were identified between 2001 and 2026 with a majority of recently published studies (**Figure 1A**). Among them, five were review articles, 31 were unrelated to the topic, 19 addressed *Achromobacter* without analyzing biofilm formation, or investigated biofilm produced by non-*Achromobacter* species and one record was a preprint duplicate of a subsequently published article (Acharya et al., 2026). These 56 studies were therefore excluded. Manual screening of the reference lists yielded ten additional studies not found with the initial search strategy (Kim et al., 2008; Frank et al., 2009; Li et al., 2013; Kampf et al., 2014; Ridderberg et al., 2015; Voronina et al., 2016; Vijay and Willcox, 2018; Dantam et al., 2020; Veschetti et al., 2020; Cao et al., 2022). Data from our recent work (Jean-Pierre et al., 2026) were also integrated. This process led to 79 studies being included in the final scoping review (**Figure 1B**), and the list of studies and their main characteristics are summarized in **Supplementary Table 1**.

Isolates reported in multiple publications were included only once in the analyses to prevent overrepresentation and ensure the independence of observations [ (Liaqat and Sabri, 2009) and (Liaqat et al., 2010); (Voronina et al., 2016) and (Li et al., 2013) (Jakobsen et al., 2013), (Cameron et al., 2019); (Nielsen et al., 2017) and (Nielsen et al., 2019); (Vandeplassche et al., 2017) and (Vandeplassche et al., 2020); (Sandri et al., 2022) and (Sandri et al., 2023); (Menetrey et al., 2020) and (Besse et al., 2025)]. Conversely, when overlap between strain collections was suspected but not explicitly documented, all isolates were retained, although the potential sharing of isolates could not be formally excluded [ (Azevedo et al., 2014) and (Azevedo et al., 2016); (Aiyer et al., 2021) and (Aiyer et al., 2022); (Mataracı-Kara et al., 2025) and (Damar-Çelik et al., 2021)]. Across these studies, we identified at least 905 *Achromobacter* strains, although in 13 publications the exact number could not be determined due to incomplete reporting. The number of isolates per study was highly variable, with a median of two (range: 1-122) (Pereira et al., 2017). The dataset was markedly dominated by CF-derived isolates (n = 691, 35 studies), highlighting the historical research focus on CF airway infections. In contrast, non-CF clinical isolates accounted for a smaller proportion (n = 156, 26 studies), including four strains recovered from biological materials or medical devices. Environmental isolates remained underrepresented (n = 39, 24 studies), despite the ecological relevance of the genus, and only two of the 905 strains had an unspecified origin (Yamasaki et al., 2018; Ebaid et al., 2025). Additionally, 17 type strains were included. Overall, the 79 studies collectively investigated 12 distinct *Achromobacter* species, although species assignment was inconsistently performed across studies.

An UpSet plot (**Figure 2**) was used to present study distribution between thematic categories identified from the literature and used for structuring this scoping review, *i.e*.: studies on molecular mechanisms of biofilm formation (“Molecular”, n = 11), on biofilm formation according to species (“Species”, n = 3), or strain origin (“Origin”, n = 3), on modification of biofilm formation during polymicrobial interaction (“Interaction”, n = 12), and finally on antibiofilm effects of antibiotics (“Antibiotic”, n = 20), or non-antibiotic strategies (“Non-antibiotic”, n = 20). Because several studies addressed multiple themes, their intersections were quantified and represented using an UpSet plot to illustrate co-occurrence patterns (Lex et al., 2014; Conway et al., 2017).

**Figure 2:**
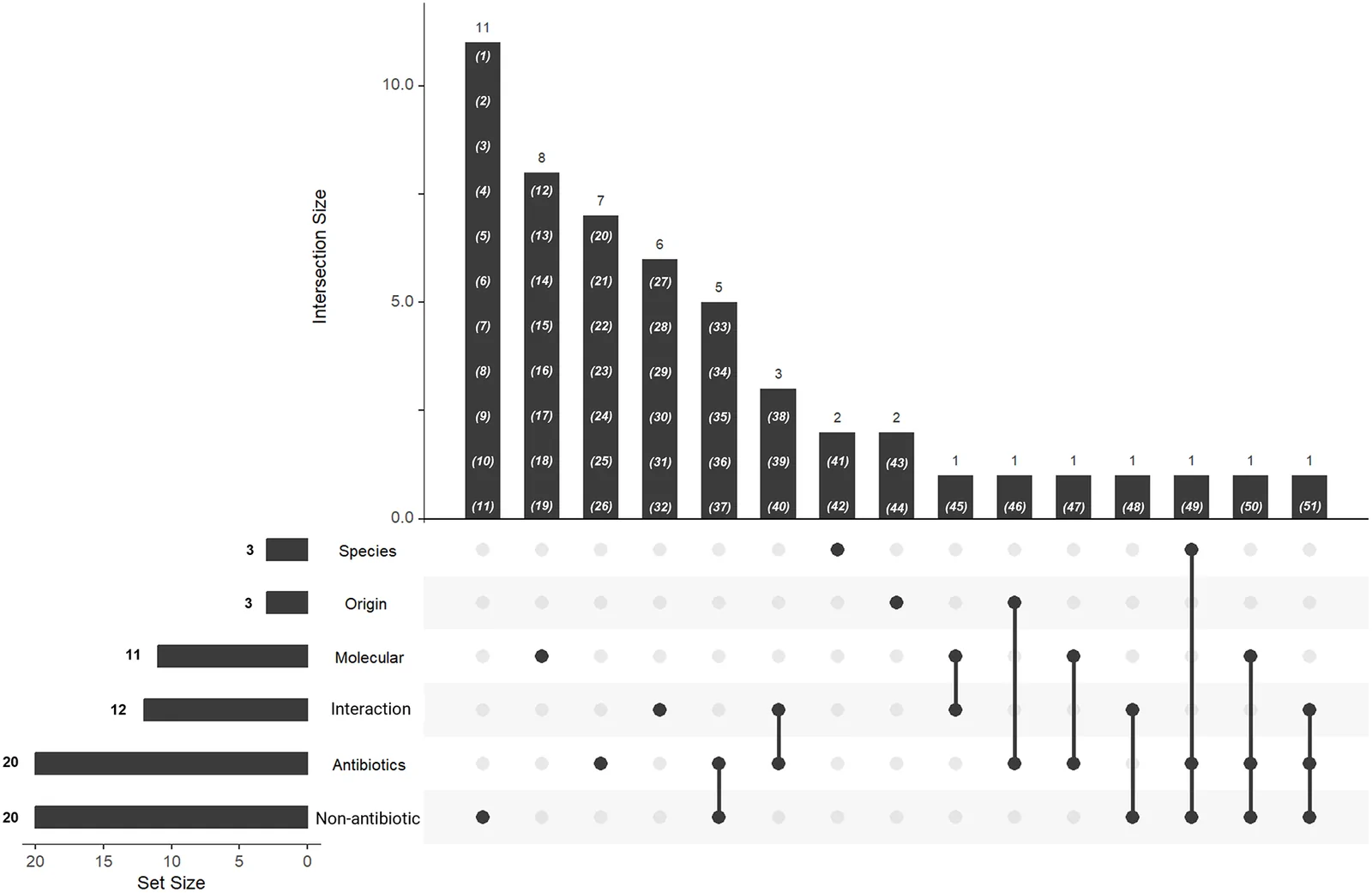
UpSet plot of thematic categories addressed in the *Achromobacter* biofilm literature. Studies were grouped into six categories: Molecular (n = 11), Species (n = 3), Origin (n = 3), Interaction (n = 12), Antibiotic (n = 20), and Non-antibiotic (n = 20). The plot displays both the number of studies within each category and the intersections between categories, illustrating the extent of thematic overlap across the literature. The references of the included studies are indicated as italicized numbers in parentheses within the corresponding bar plots: *1* (LiPuma et al., 2009); *2* (Danilova et al., 2022); *3* (McBain et al., 2003); *4* (Flores-Maldonado et al., 2025); *5* (Hugon et al., 2015); *6* (Montoya-Hinojosa et al., 2023); *7* (De Souza Freitas et al., 2020); *8* (Chang et al., 2015); *9* (Yamasaki et al., 2018); *10* (Liaqat et al., 2010); *11* (Kampf et al., 2014); *12* (Sato et al., 2026); *13* (Khademi et al., 2021); *14* (Al-Kahachi et al., 2023); *15* (Jakobsen et al., 2013); *16* (Voronina et al., 2016); *17* (Ridderberg et al., 2015); *18* (Li et al., 2013); *19* (Nielsen et al., 2019); *20* (Tom et al., 2016); *21* (Liaqat et al., 2009); *22* (Ulloa et al., 2020); *23* (Wan et al., 2025); *24* (Wackernagel et al., 2025); *25* (Mataracı-Kara et al., 2025); *26* (Montoya-Hinojosa et al., 2024); *27* (Sahl et al., 2025); *28* (Besse et al., 2025); *29* (Frank et al., 2009); *30* (Menetrey et al., 2020); *31* (Azevedo et al., 2014); *32* (Abdouchakour et al., 2015); *33* (Flores-Ibarra et al., 2026); *34* (Ebaid et al., 2025); *35* (Damar-Çelik et al., 2021); *36* (Aiyer et al., 2022); *37* (Aiyer et al., 2021); *38* (Azevedo et al., 2016); *39* (Vandeplassche et al., 2017); *40* (Vandeplassche et al., 2020); *41* (Sahl et al., 2023); *42* (Pereira et al., 2017); *43* (Filipic et al., 2017); *44* (Acharya et al., 2026); *45* (Sandri et al., 2021); *46* (Jean-Pierre et al., 2026); *47* (Cameron et al., 2019); *48* (Bellavita et al., 2023); *49* (Nielsen et al., 2016); 5*0* (Nielsen et al., 2017); *51* (Yilmaz et al., 2023).

The substantial heterogeneity in study designs and methodologies precluded formal meta-analyses. However, we considered data from studies using the same biofilm assessment method and interpretative criteria to be comparable for statistical analysis. According to the Stepanović classification, mean optical density (OD) values are calculated from replicate measurements of each strain and the negative control, and the cut-off value (ODc) is defined as the mean OD of the negative control plus three standard deviations. Isolates are then classified according to their OD relative to ODc as non-biofilm producers (OD ≤ ODc), weak (ODc < OD ≤ 2 × ODc), moderate (2 × ODc < OD ≤ 4 × ODc), or strong (OD > 4 × ODc) biofilm producers (Stepanović et al., 2000, 2007). These standardized criteria ensured that comparable data could be pooled into contingency tables for statistical analyses according to species and isolate origin.

## 3 Molecular insights into biofilm formation and regulation in *Achromobacter*

To date, 11 studies have investigated the molecular basis of biofilm development in *Achromobacter*, collectively analyzing 109 non-duplicate isolates from nine species, with *A. xylosoxidans* overwhelmingly predominant (69% of the strains), and two isolates with undefined species assignment (Jakobsen et al., 2013; Li et al., 2013; Ridderberg et al., 2015; Voronina et al., 2016; Nielsen et al., 2017, 2019; Cameron et al., 2019; Khademi et al., 2021; Al-Kahachi et al., 2023; Sato et al., 2026; Veschetti et al., 2026). Among these, Nielsen et al.’s transcriptomic and functional work on a CF-derived *A. xylosoxidans* isolate remains the most comprehensive characterization available (Nielsen et al., 2019). Key findings from these studies are summarized in **Figure 3**.

**Figure 3:**
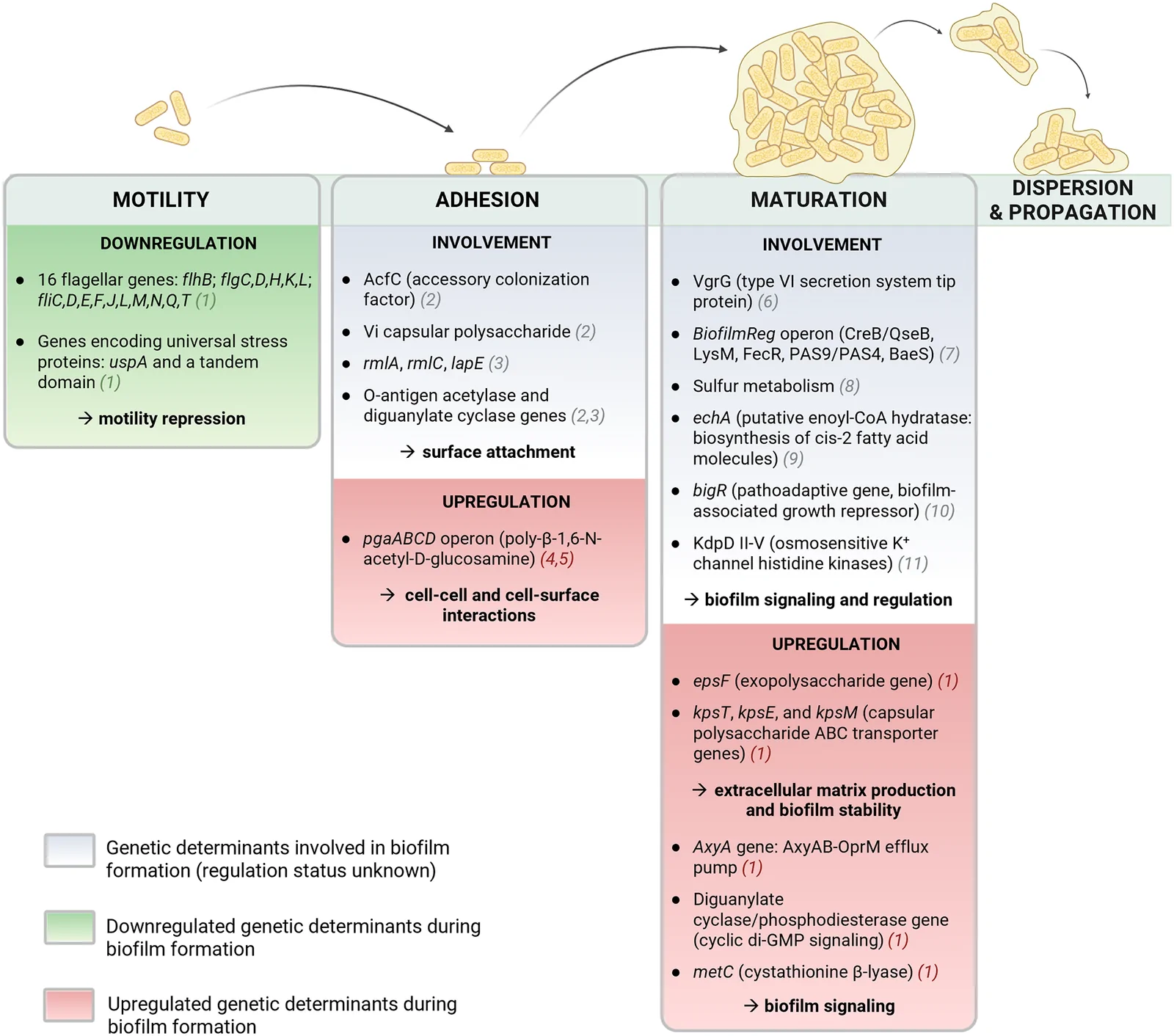
Life cycle of *Achromobacter* biofilm and associated genetic determinants. The references are indicated by italicized numbers in parentheses: *1* (Nielsen et al., 2019); *2* (Li et al., 2013); *3* (Ridderberg et al., 2015); *4* (Jakobsen et al., 2013); *5* (Sato et al., 2026); *6* (Sandri et al., 2021); *7* (Voronina et al., 2016); *8* (Nielsen et al., 2017); *9* (Cameron et al., 2019); *10* (Khademi et al., 2021); *11* (Al-Kahachi et al., 2023).

### 3.1 Transition from planktonic to sessile growth: motility repression

Flagellar motility plays an important role during the early stages of biofilm formation by facilitating initial surface colonization. Consistent with this role, deletion of *fliF*, encoding the flagellar M-ring protein, reduced *in vitro* biofilm formation by 39% (Nielsen et al., 2019). However, once attachment has occurred, the transition from planktonic to biofilm-associated growth involves extensive transcriptional reprogramming, particularly within motility-related pathways (Nielsen et al., 2019). Sixteen flagellar genes (*flhB*; *flgC*,*D*,*H*,*K*,*L*; *fliC*,*D*,*E*,*F*,*J*,*L*,*M*,*N*,*Q*,*T*) are markedly downregulated, reflecting the reduced motility of the sessile state. Concomitantly, genes encoding universal stress proteins (*uspA* and a tandem-domain protein) are also downregulated, a pattern that may contribute to motility repression and promote surface-associated growth (**Figure 3**).

### 3.2 Adhesion and early biofilm formation

Following motility repression, surface attachment is mediated by a diverse repertoire of adhesins, cell-envelope components, and matrix-associated determinants. The *pgaABCD* operon, encoding poly-β-1,6-*N*-acetyl-D-glucosamine, promotes both cell-cell and cell-surface adhesion (Jakobsen et al., 2013) and its upregulation has been associated with enhanced biofilm formation in response to extracellular signals (Sato et al., 2026). Additional adhesion-related genes include *rmlA*, *rmlC*, and *lapE* genes encoding O-antigen acetylase and diguanylate cyclase (Li et al., 2013; Ridderberg et al., 2015). They contribute to surface interaction and potential matrix modifications. Moreover, other capsular components, including the Vi capsular polysaccharide, and the accessory colonization factor AcfC further support adhesion, environmental persistence, and host interaction (Li et al., 2013). Finally, a recent study identified a full-length aspartate kinase as a potential determinant of the biofilm-forming phenotype, as biofilm-producing isolates predominantly harbored the complete gene, while non-producers mainly carried a variant lacking part of the ATP-binding domain (Veschetti et al., 2026) (**Figure 3**).

### 3.3 Biofilm maturation and matrix development

As adhesion progresses, *Achromobacter* initiates transcriptional programs supporting matrix synthesis and biofilm stability. This part is characterized by the upregulation of genes involved in exopolysaccharide (EPS) biosynthesis (e.g., *epsF*) and cell envelope biogenesis, including capsular ABC transporters (*kpsT, kpsE*, *kpsM*). The KpsT/KpsM complex facilitates polysaccharide export across the cytoplasmic membrane, thereby contributing to extracellular matrix production and biofilm stability (Nielsen et al., 2019). Biofilm-associated growth is also characterized by increased expression of the AxyAB-OprM efflux pump, suggesting roles beyond antimicrobial resistance, possibly including the export of metabolites or signaling molecules relevant to biofilm physiology. In addition, enhanced expression of a diguanylate cyclase/phosphodiesterase gene supports the involvement of cyclic di-GMP as a key second messenger in *Achromobacter* biofilm development. In parallel, strong overexpression of *metC* (cystathionine β-lyase) indicates remodeling of the sulfur metabolism, probably reflecting adaptation to oxygen and nutrient limitation within the maturing biofilm (Nielsen et al., 2019).

Biofilm maturation involves several regulatory systems. The *BiofilmReg* operon, identified by Voronina et al., encodes a two-component regulatory system (CreB/QseB), a LysM-FecR domain protein, and a periplasmic sensor histidine kinase (PAS9/PAS4, BaeS). This conserved cluster - whose phylogeny suggests horizontal acquisition from *Burkholderia* - plays a central role in coordinating *Achromobacter* biofilm formation (Voronina et al., 2016). Beyond this pathway, Cameron et al. demonstrated that *echA*, encoding a putative enoyl-CoA hydratase involved in diffusible signaling factor synthesis, shaped biofilm architecture in *A. xylosoxidans* (Cameron et al., 2019). Additional regulatory pathways may also contribute to biofilm formation in *Achromobacter*. These include several osmosensitive K^+^-channel histidine kinases (KdpD II-V) which may participate in electrical or osmotic signaling within biofilm (Al-Kahachi et al., 2023), the type VI secretion system tip protein VgrG involved in interspecies competition, as well as modulation of biofilm formation (Sha et al., 2013), and the pathoadaptive gene *bigR*, a biofilm-associated growth repressor (Khademi et al., 2021) (**Figure 3**).

Together, these findings highlight the diversity of regulatory pathways involved in *Achromobacter* biofilm formation and maturation.

### 3.4 Adaptation during persistent host colonization

Transcriptomic data, currently available only for longitudinal CF isolates, reinforce mechanistic insights regarding *Achromobacter* biofilm formation. During chronic lung colonization, *Achromobacter* exhibits efflux pump upregulation, which is consistent with greater antibiotic tolerance and downregulation of motility genes, which reflects decreased motility. Furthermore, it exhibits enhanced expression of genes involved in sulfur, protein, and potassium metabolism, which indicates metabolic remodeling to cope with the microaerobic, nutrient-limited and osmotic stresses encountered in the CF lung (Nielsen et al., 2017).

Together, these adaptations mirror the transcriptional trajectories observed during *in vitro* biofilm maturation, suggesting strong crosstalk between biofilm physiology and chronic infection dynamics.

## 4 Methodological diversity and challenges in studying the biofilm-forming capacity of *Achromobacter* spp.

### 4.1 Diversity of experimental methods used to study *Achromobacter* biofilm

The studies we reviewed revealed substantial methodological heterogeneity in how *Achromobacter* biofilm was investigated. According to the classification proposed by Azeredo et al. (Azeredo et al., 2017), methods included chemical approaches (n = 51), such as crystal violet (CV) staining to estimate total mature biofilm biomass without distinguishing between viable and non-viable cells; microbiological approaches (n = 29), including colony-forming unit (CFU) enumeration to quantify viable bacteria within mature biofilms; microscopy-based approaches (n = 24), such as confocal laser scanning microscopy and scanning electron microscopy to characterize biofilm architecture and spatial organization; molecular approaches (n = 19), targeting biofilm-associated genes or gene expression; and physical approaches (n = 3), including the Biofilm Ring Test^®^ (BioFilm Control, Saint-Beauzire, France), which assesses the early stages of biofilm formation, particularly bacterial adhesion. The advantages and limitations of these different approaches have been reviewed in detail elsewhere (Azeredo et al., 2017). These methods were predominantly applied under *in vitro* conditions (n = 62), although *ex vivo* investigations (n = 15) were also conducted directly on clinical materials or tissue specimens. In contrast, *in vivo*/clinical demonstration of *Achromobacter* biofilm formation remains scarce, with only two studies reported to date (Wackernagel et al., 2025; Wan et al., 2025). An overview of this methodological diversity is illustrated in **Figure 4**. CV staining, an indirect method used to quantify total biofilm biomass, was the most commonly employed technique in *Achromobacter* biofilm studies, being reported in 63.3% (50/79) of the reviewed publications. However, despite its widespread use, CV staining was performed using non-standardized protocols across the reviewed studies. Interpretation criteria varied widely, with biofilm production assessed, when reported, either according to the Stepanović criteria (Stepanović et al., 2000, 2007) or reported relative to the reference strain *P. aeruginosa* PaO1 only (Jakobsen et al., 2013; Nielsen et al., 2016, 2017; Sahl et al., 2023, 2025; Acharya et al., 2026), resulting in substantial methodological heterogeneity and limiting comparisons across studies. We therefore conducted focused analyses in the subsequent subsections of this scoping review, prioritizing results from the most frequently used CV-based studies applying the Stepanović interpretative criteria, in order to enable statistical comparisons across comparable datasets.

**Figure 4:**
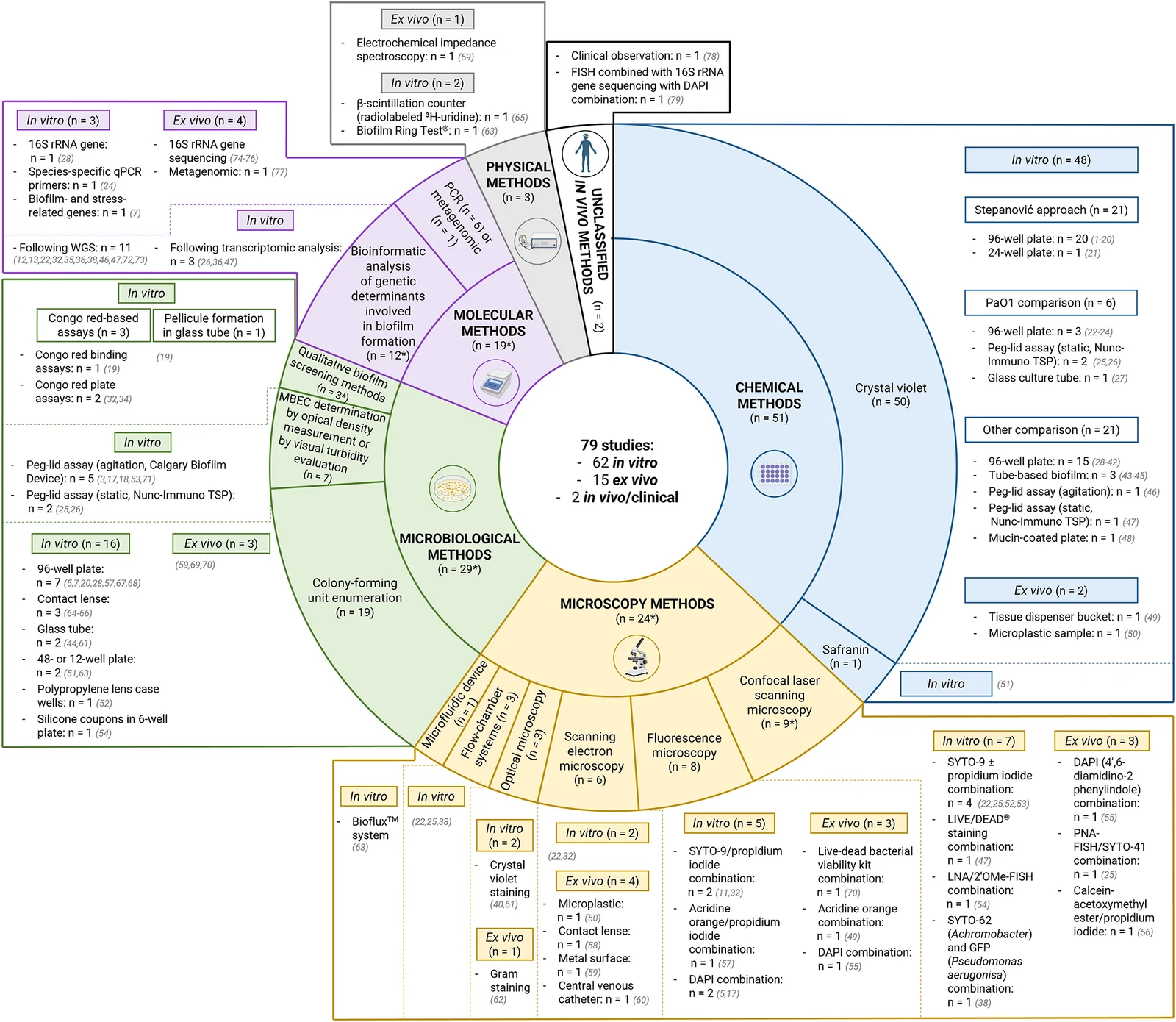
Diversity of experimental methods used to study *Achromobacter* biofilm. *indicates the number of different studies that used this method. DAPI, 4′,6-diamidino-2-phenylindole; FISH, fluorescence *in situ* hybridization; MBEC, minimum biofilm eradication concentration; n, number of publications; qPCR, quantitative real-time PCR; WGS, whole-genome sequencing. References are abbreviated as italicized numbers in the table: *1* (Abdouchakour et al., 2015); *2* (Firmida et al., 2017); *3* (Montoya-Hinojosa et al., 2024); *4* (Mataracı-Kara et al., 2025); *5* (Azevedo et al., 2014); *6* (Menetrey et al., 2020); *7* (Bellavita et al., 2023); *8* (Sandri et al., 2022); *9* (Sandri et al., 2023); *10* (Besse et al., 2025); *11* (Damar-Çelik et al., 2021); *12* (Khademi et al., 2021); *13* (Veschetti et al., 2026); *14* (Trancassini et al., 2014); *15* (Filipic et al., 2017); *16* (Liu et al., 2016); *17* (Flores-Ibarra et al., 2026); *18* (Montoya-Hinojosa et al., 2023); *19* (Lazzem et al., 2023); *20* (LiPuma et al., 2009); *21* (Konstantinović et al., 2017); *22* (Jakobsen et al., 2013); *23* (Sahl et al., 2023); *24* (Sahl et al., 2025); *25* (Nielsen et al., 2016); *26* (Nielsen et al., 2017); *27* (Acharya et al., 2026); *28* (Vandeplassche et al., 2020); *29* (Kampf et al., 2014); *30* (Veschetti et al., 2020); *31* (Liaqat et al., 2010); *32* (Cameron et al., 2019); *33* (Pereira et al., 2017); *34* (Ebaid et al., 2025); *35* (Al-Kahachi et al., 2023); *36* (Sato et al., 2026); *37* (De Souza Freitas et al., 2020); *38* (Sandri et al., 2021); *39* (Flores-Maldonado et al., 2025); *40* (Soon et al., 2025); *41* (Zahra et al., 2023); *42* (Danilova et al., 2022); *43* (Yadav et al., 2023); *44* (Liaqat et al., 2009); *45* (Kapadia et al., 2022); *46* (Ridderberg et al., 2015); *47* (Nielsen et al., 2019); *48* (Aiyer et al., 2022); *49* (Günther et al., 2016); *50* (Deng et al., 2024); *51* (Ulloa et al., 2020); *52* (Chang et al., 2015); *53* (Tom et al., 2016); *54* (Azevedo et al., 2016); *55* (Frank et al., 2009); *56* (Cao et al., 2022); *57* (Yilmaz et al., 2023); *58* (Wiley et al., 2012); *59* (Narenkumar et al., 2019); *60* (Kim et al., 2008); *61* (Marion-Sanchez et al., 2020); *62* (Hansen et al., 2010); *63* (Jean-Pierre et al., 2026); *64* (Vijay and Willcox, 2018); *65* (Dantam et al., 2020); *66* (Yamasaki et al., 2018); *67* (Vandeplassche et al., 2017); *68* (Aiyer et al., 2021); *69* (Hennebique et al., 2024); *70* (McBain et al., 2003)*; 71* (Hugon et al., 2015); *72* (Li et al., 2013); *73* (Voronina et al., 2016); *74* (Liaqat and Sabri, 2009); *75* (Choe et al., 2012); *76* (Choe et al., 2013); *77* (Sadia et al., 2026); *78* (Wan et al., 2025); *79* (Wackernagel et al., 2025).

### 4.2 Biofilm formation by the *A. xylosoxidans* type strain: a paradigm of method-dependent variability

To date, 13 studies have investigated the biofilm-forming capacity of the *A. xylosoxidans* type strain (ATCC 27061 (Chang et al., 2015; Dantam et al., 2020; Aiyer et al., 2021, 2022; Yilmaz et al., 2023) = LMG 1863 (Nielsen et al., 2016; Damar-Çelik et al., 2021) = KM543 (Sahl et al., 2025) = CIP 71.32 (Marion-Sanchez et al., 2020) = CCUG-56438 (Sahl et al., 2023) = DSM 2402 (Jakobsen et al., 2013; Bellavita et al., 2023) = NCTC 10807 (Voronina et al., 2016)). Depending on the model, quantification method, and interpretation criteria used, conclusions differed strikingly, ranging from non-producer to strong biofilm-producer. Using a continuous-flow model, Nielsen et al. showed that the *A. xylosoxidans* type strain can form surface-attached biofilm consisting of 5-10 µm spherical aggregates (Nielsen et al., 2016). Biofilm formation has also been documented on contact lens materials (Chang et al., 2015), with strong adherence demonstrated to hydrogel (etafilcon A) and silicone hydrogel (senofilcon A) lenses, although viability was lower on the latter (Dantam et al., 2020). A demonstration of long-term persistence was provided by Marion-Sanchez et al., who showed that the type strain remained capable of forming biofilm for up to two years when incubated in glass tubes containing dialysis or microfiltered water at 30 °C, though not in distilled water, using CV staining and surface-coverage microscopy (Marion-Sanchez et al., 2020). Several studies confirmed biofilm formation using viable cell enumeration (CFU/mL) in mature biofilm (Aiyer et al., 2021; Yilmaz et al., 2023), and CV staining assays (Jakobsen et al., 2013; Nielsen et al., 2016; Aiyer et al., 2022; Bellavita et al., 2023). However, CV assays produced inconsistent categorizations depending on the interpretation criteria used, identifying the type strain either as a weak or non-producer (Damar-Çelik et al., 2021; Sahl et al., 2025) or, conversely, as a strong biofilm producer (Jakobsen et al., 2013; Bellavita et al., 2023).

Taken together, these discrepancies illustrate the profound influence of the experimental model (static *versus* flow systems; abiotic surface type; medium composition), the scoring approach (CV staining, CFU enumeration, microscopy-based quantification), and the interpretation criteria (quantitative thresholds, Stepanović classification) on the assessment of *Achromobacter* biofilms. Such methodological variability is not unique to *Achromobacter* and has been widely reported for other bacterial pathogens (Peeters et al., 2008; Stiefel et al., 2016). While these observations underscore the need for greater methodological harmonization when comparing biofilm phenotypes, they also highlight the complementary nature of different biofilm assays, each providing distinct insights into biofilm formation and structure (Azeredo et al., 2017).

## 5 Identification challenges and *Achromobacter* species biofilm-formation ability

### 5.1 Reliability and limitations of species-level identification in *Achromobacter* biofilm studies

The *Achromobacter* genus currently includes 22 validly described species, with *A. xylosoxidans* being the most frequently recovered from human infections - both in CF and non-CF contexts - suggesting that it may harbor specific traits that contribute to its epidemiological success (Sorlin et al., 2024). However, species-level identification remains a major blockage in *Achromobacter* research and strongly influences the interpretation of biofilm-related findings. Routine diagnostic tools such as matrix-assisted laser desorption ionization-time of flight mass spectrometry (MALDI-ToF MS), reliably identify *Achromobacter* at genus level, but lack discriminatory accuracy at species level due to incomplete reference spectral databases (Veschetti et al., 2022). As a result, definitive species assignment relies on sequencing-based approaches, including *nrdA* gene sequencing (Spilker et al., 2013), multi-locus sequence typing (MLST) (Spilker et al., 2012), and whole-genome sequencing (Isler et al., 2020). Despite their reliability, these methods remain infrequently applied, limiting the ability to interpret species-specific phenotypes, particularly regarding biofilm formation. Among the 79 studies included in this scoping review, nine did not report the identification method used, and 32 relied on inadequate or unreliable approaches for species-level assignment, representing a total of 291 strains, which were therefore reported as *Achromobacter* sp. These included 16S rRNA gene sequencing (22/32), MALDI-ToF MS (8/32), *recA* gene sequencing (3/32), API^®^ 20NE identification kits (2/32), VITEK^®^ 2 Gram-Negative identification card (2/32), a species-specific 163-bp PCR assay (Trancassini et al., 2014), and standard clinical microbiological workflows (Kim et al., 2008). In addition, four studies reported misidentified strains. Strain ATCC 13138 was initially described as *A. xylosoxidans*, although it corresponds to *Achromobacter mucicolens* in the DSMZ database (De Souza Freitas et al., 2020). Likewise, strain LMG 26680 was reported as *A. xylosoxidans* but is listed as *Achromobacter denitrificans* in the PubMLST database (Vandeplassche et al., 2017, 2020). Similarly, strain AXX-A was identified as *A. xylosoxidans* despite being classified as *Achromobacter insuavis*, and *A. piechaudii* HLE corresponds to *Achromobacter marplatensis* according to the PubMLST and/or NCBI databases (Li et al., 2013). These discrepancies highlight the central importance of robust species identification in all studies on *Achromobacter*, including those on biofilm formation.

To ensure reliability, subsequent analysis focusing on species-specific biofilm formation was restricted to 38 studies that employed validated species-level identification methods. These studies, representing 620 strains, included strains that were reclassified following database verification (Li et al., 2013; Vandeplassche et al., 2017, 2020; De Souza Freitas et al., 2020). Among these, whole-genome sequencing was used in 16 studies (Jakobsen et al., 2013; Li et al., 2013; Ridderberg et al., 2015; Voronina et al., 2016; Cameron et al., 2019; Nielsen et al., 2019; Veschetti et al., 2020, 2026; Khademi et al., 2021; Sandri et al., 2021, 2023; Al-Kahachi et al., 2023; Besse et al., 2025; Sahl et al., 2025; Acharya et al., 2026; Sato et al., 2026), encompassing 154 strains, including one using pyrosequencing (GS FLX Titanium) (Jakobsen et al., 2013), 11 using Illumina technology, one combining Nanopore and Illumina sequencing (Acharya et al., 2026), and one using the PacBio Revio platform (Sato et al., 2026) whereas two studies did not specify the sequencing technology used. One additional study employed shotgun metagenomic sequencing (Sadia et al., 2026). MLST provided the highest-resolution approach in seven studies (206 strains), whereas *nrdA* allele sequencing served as the primary identification method in six studies (252 strains). Finally, six studies relied exclusively on type strains (six strains).

### 5.2 Biofilm production according to species in the *Achromobacter* genus

Biofilm-forming ability has been investigated in 12 *Achromobacter* species. Only four species are represented by more than ten strains: *A. xylosoxidans* (n = 428), *Achromobacter ruhlandii* (n = 59) (Ridderberg et al., 2015; Nielsen et al., 2016; Voronina et al., 2016; Pereira et al., 2017; Damar-Çelik et al., 2021; Sahl et al., 2023; Mataracı-Kara et al., 2025; Sadia et al., 2026), *A. insuavis* (n = 55) (Li et al., 2013; Ridderberg et al., 2015; Nielsen et al., 2016; Filipic et al., 2017; Veschetti et al., 2020, 2026; Damar-Çelik et al., 2021; Sandri et al., 2021; Sahl et al., 2023; Mataracı-Kara et al., 2025), and *A. dolens* (n = 12) (Damar-Çelik et al., 2021; Sahl et al., 2023; Mataracı-Kara et al., 2025; Veschetti et al., 2026). The remaining species are only sparsely represented: *A. aegrifaciens* (n = 6) (Damar-Çelik et al., 2021; Sandri et al., 2023; Mataracı-Kara et al., 2025; Veschetti et al., 2026), *A. piechaudii* (n = 5) (Li et al., 2013; Filipic et al., 2017; Damar-Çelik et al., 2021; Mataracı-Kara et al., 2025), *A. mucicolens* (n = 4) (De Souza Freitas et al., 2020; Damar-Çelik et al., 2021; Al-Kahachi et al., 2023; Mataracı-Kara et al., 2025), *A. denitrificans* (n = 3) (Vandeplassche et al., 2017, 2020; Damar-Çelik et al., 2021; Mataracı-Kara et al., 2025), *A. spiritinus* (n = 3) (Filipic et al., 2017; Damar-Çelik et al., 2021; Mataracı-Kara et al., 2025), *A. insolitus* (n = 2) (Filipic et al., 2017; Sandri et al., 2023; Sadia et al., 2026), *A. arsenitoxydans* (n = 1) (Li et al., 2013), and *A. marplatensis* (n = 1) (Li et al., 2013). Additional isolates are reported as *Achromobacter dolens/A. ruhlandii* (n = 4) (Filipic et al., 2017), as *Achromobacter* sp. of unspecified genogroup (n = 5) (Damar-Çelik et al., 2021; Mataracı-Kara et al., 2025), or simply as *Achromobacter* sp. (n = 33) (Abdouchakour et al., 2015; Liu et al., 2016; Sandri et al., 2023; Veschetti et al., 2026).

Among species, *A. xylosoxidans* is the most extensively studied and consistently emerges as the strongest biofilm-former. Using the CV assay interpreted with Stepanović classification (Stepanović et al., 2000, 2007) (**Table 1**), 37.7% of *A. xylosoxidans* strains (54/143) were categorized as strong biofilm producers across several independent studies (Filipic et al., 2017; Firmida et al., 2017; Menetrey et al., 2020; Damar-Çelik et al., 2021; Bellavita et al., 2023; Sandri et al., 2023; Mataracı-Kara et al., 2025). Although strong biofilm producers have been described in other taxa, such as one single *A. aegrifaciens* strain (Sandri et al., 2023), one single *A. dolens*/*A. ruhlandii* strain (Filipic et al., 2017) and three strains of an unspecified *Achromobacter* genogroup (Damar-Çelik et al., 2021; Mataracı-Kara et al., 2025), these remain rare observations (**Table 1**).

**Table 1:** Biofilm production by crystal violet assay (Stepanović classification) according to *Achromobacter* species*^a^* (statistical analyses combining the results of these independent studies are presented in the text).

Species (or genogroup)	Reference	No of strains	Non BP	Weak BP	Moderate BP	Strong BP
*A. aegrifaciens*	*1*	2	1	–	1	–
	*2*	1	1	–	–	–
	*3*	1	–	–	–	1
	Total	4	2	-	1	1
	*4^b^*	2	1	1*^b^*		
*A. denitrificans*	*1*	1	1	–	–	–
	*2*	1	1	–	–	–
	Total	2	2	-	-	-
*A. dolens*	*1*	1	1	–	–	–
	*2*	1	–	1	–	–
	Total	2	1	1	-	-
	*4^b^*	6	5	1*^b^*		
*A. dolens/* *A. ruhlandii*	*5*	4	1	1	1	1
	Total	4	1	1	1	1
Unspecified *A.* genogroup	*1*	3	1	–	–	2
	*2*	2	1	–	–	1
	Total	5	2	-	-	3
*A. insolitus*	*3*	1	–	–	1	–
	*5*	1	–	–	1	–
	Total	2	-	-	2	-
*A. insuavis*	*1*	11	11	–	–	–
	*2*	10	10	–	–	–
	*5*	2	–	2	–	–
	Total (%)	23 (100)	21 (91.3)	2 (8.7)	-	-
	*4^b^*	4	2	2*^b^*		
*A. mucicolens*	*1*	1	1	–	–	–
	*2*	1	1	–	–	–
	Total	2	2	-	-	-
*A. piechaudii*	*1*	1	–	–	1	–
	*2*	2	–	–	2	–
	*5*	1	–	1	–	–
	Total	4	-	1	3	-
*A. ruhlandii*	*1*	9	8	1	–	–
	*2*	9	8	1	–	–
	Total (%)	18 (100)	16 (88.9)	2 (11.1)	-	-
*A. spiritinus*	*1*	1	1	–	–	–
	*2*	1	1	–	–	–
	*5*	1	1	–	–	–
	Total	3	3	-	-	-
*A. xylosoxidans*	*1*	22	12	7	–	3
	*2*	24	12	9	–	3
	*3*	5	–	3	–	2
	*5*	60	–	12	29	19
	*6*	28	–	4	–	24
	*7*	3	–	–	1	2
	*8*	1	–	–	–	1
	Total (%)	143 (100)	24 (16.8)	35 (24.5)	30 (21)	54 (37.7)
	*4^b^*	8	3	5*^b^*		
	*9^b^*	55	40	15*^b^*		
OVERALL (%)		287 (100)	125 (43.5)	42 (≥14.6)*^c^*	37 (≥12.9)*^c^*	59 (≥20.6)*^c^*

Percentages were calculated and reported in parentheses only when the total number of strains exceeded 10. Statistical analysis is presented in the main text.

^a^ scoping review limited to studies with accurate species identification;.

^b^ biofilm production was reported as a binary outcome, without classification into weak, moderate, or strong producers: the values were highlighted in light gray to indicate that they encompass all three categories;

^c^ excluding the biofilm-producing strains that were not classified into weak, moderate, or strong producers (Khademi et al., 2021; Veschetti et al., 2026). References are abbreviated as italicized numbers: *1* (Damar-Çelik et al., 2021); *2* (Mataracı-Kara et al., 2025); *3* (Sandri et al., 2023); *4* (Veschetti et al., 2026); *5* (Filipic et al., 2017); *6* (Firmida et al., 2017); *7* (Menetrey et al., 2020); *8* (Bellavita et al., 2023); *9* (Khademi et al., 2021). A dash (-) indicates that no isolates were reported. BP, biofilm producer; No, number.

After *A. xylosoxidans*, *A. insuavis* is the second most commonly studied species using this methodology (Stepanović et al., 2000, 2007). Among the 27 strains assessed with the Stepanović CV assay, 23 were classified as non-biofilm producers (Damar-Çelik et al., 2021; Mataracı-Kara et al., 2025; Veschetti et al., 2026) (**Table 1**). Poor adhesion has been documented on abiotic surfaces such as polystyrene (Veschetti et al., 2020) and glass (Sandri et al., 2021), characterized by sporadic, unstable, and loosely attached aggregates. Under flow conditions, an *A. insuavis* strain (CF4-b) failed to form surface-attached biofilm, instead producing a loosely attached or unattached biofilm composed of large, suspended aggregates (up to 80 µm) (Nielsen et al., 2016). However, other exceptions have been reported. In the same study, another *A. insuavis* strain (CF5-a) produced substantial attached biofilm, comparable to *P. aeruginosa* PaO1 (Nielsen et al., 2016) and all nine clinical isolates tested by peg-lid CV assay were capable of forming biofilm, highlighting strong methodological dependence on biofilm assessment.

*A. ruhlandii* represents the third most studied species using Stepanović CV classification (**Table 1**): 16/18 strains were classified as non-biofilm producers and two were weak producers (Damar-Çelik et al., 2021; Mataracı-Kara et al., 2025). However, both the *A. ruhlandii* type strain (CCUG 38886^T^) and a clinical isolate (CF9) formed robust biofilm comparable to *P. aeruginosa* PaO1 (Nielsen et al., 2016). In addition, Pereira et al. reported that all *A. ruhlandii* isolates produced biofilm (n = 28) (Pereira et al., 2017) and a recent metagenomic analysis of chronic wound microbiomes revealed *A. ruhlandii* within polymicrobial biofilm communities from both calf and thigh infection sites, where it represented 20% relative abundance at each site (Sadia et al., 2026).

For less commonly studied species, the available data are sparse and sometimes contradictory. None of the two *A. mucicolens* strains evaluated using the Stepanović CV method produced detectable biofilm (Damar-Çelik et al., 2021; Mataracı-Kara et al., 2025) (**Table 1**). In contrast, biofilm formation was reported for the *A. mucicolens* ATCC 13138 strain (initially misidentified as *A. xylosoxidans*) (De Souza Freitas et al., 2020), as well as for a clinical isolate (Al-Kahachi et al., 2023). Similarly, none of the two *A. denitrificans* strains assessed using the Stepanović CV method produced biofilm (Damar-Çelik et al., 2021; Mataracı-Kara et al., 2025), whereas the *A. denitrificans* LMG 26680 strain (also initially reported as *A. xylosoxidans*) formed biofilm as demonstrated by both CV staining and CFU enumeration (Vandeplassche et al., 2020), supporting a strain-dependent phenotype (**Table 1**).

Although several studies include isolates belonging to several *Achromobacter* species (Li et al., 2013; Ridderberg et al., 2015; Nielsen et al., 2016; Voronina et al., 2016; Filipic et al., 2017; Pereira et al., 2017; Vandeplassche et al., 2017; Veschetti et al., 2020, 2026; Damar-Çelik et al., 2021; Sahl et al., 2023; Sandri et al., 2023; Mataracı-Kara et al., 2025), only three studies directly compared biofilm production between species (Nielsen et al., 2016; Pereira et al., 2017; Sahl et al., 2023). Nielsen et al. described distinct biofilm morphotypes, with two *A. xylosoxidans* isolates forming surface-attached biofilm consisting of small aggregates, whereas one single *A. insuavis* strain exhibited a loosely attached or unattached biofilm phenotype consisting of large suspended aggregates. However, the limited number of strains evaluated precluded definitive conclusions regarding species-specific traits (Nielsen et al., 2016). This interpretation was supported by Pereira et al., who found no significant differences in biofilm formation between *A. xylosoxidans* (n = 94) and *A. ruhlandii* (n = 28) (Pereira et al., 2017), and by Sahl et al., who observed no differences in biofilm biomass among *Achromobacter* species (Sahl et al., 2023).

Although these studies suggest strain-dependent rather than species-specific behavior, morphological differences were noted (e.g., filamentous *vs.* rod-shaped structures; abundance of flagella) without any clear correlation with biomass (Sahl et al., 2023).

### 5.3 Statistical-analyses of reviewed data highlight a significant predominance of biofilm-producing strains in *A. xylosoxidans*

Our statistical analysis restricted to CV-based studies applying the Stepanović classification (**Table 1**), revealed a significant association between biofilm formation and species. Overall, 67.5% of *A. xylosoxidans* strains (139/206) produced biofilm, compared with 28.4% of strains from other *Achromobacter* species (23/81) (χ^2^ test, *p* < 0.0001). Among biofilm-producing strains, strong biofilm production was observed in 54/119 (45.4%) *A. xylosoxidans* isolates and 5/19 (26.3%) isolates belonging to other species, with no statistically significant difference between groups (χ^2^ test, *p* = 0.1188), likely reflecting limited statistical power due to the small number of biofilm-producing isolates from non-*xylosoxidans* species. Species-level comparisons restricted to taxa represented by more than ten strains (*A. insuavis* and *A. ruhlandii*) confirmed these trends (χ^2^ test, *p* < 0.0001). *Post-hoc* pairwise comparisons using Bonferroni correction showed a significantly higher proportion of biofilm-producing isolates in *A. xylosoxidans* compared with *A. insuavis* (139/206 *vs.* 4/27, *p* < 0.0001) and *A. ruhlandii* (139/206 *vs.* 2/18, *p* < 0.0001), whereas no difference was detected between *A. insuavis* and *A. ruhlandii* (4/27 *vs.* 2/18, *p* = 1.0). Importantly, strong biofilm production was observed exclusively in *A. xylosoxidans* (54/119, 45.4%), with none of the *A. insuavis* (0/2) or *A. ruhlandii* (0/2) isolates classified as strong producers, supporting the need for additional studies to determine whether *A. xylosoxidans* specifically exhibits a higher proportion of strong biofilm producers.

## 6 Strain-dependent rather than origin-related biofilm formation in *Achromobacter*

The studies included in this scoping review investigated biofilm formation by *Achromobacter* in diverse settings, including CF-related contexts (35 studies), non-CF clinical contexts (26 studies), and environmental sources (24 studies) (**Supplementary Table 1**). Given the recognized association between CF and biofilm-related infections (Høiby, 2017; Vestby et al., 2020; Gondil and Subhadra, 2023), we assessed whether bacterial source influences biofilm-forming capacity, particularly in CF-associated contexts. Although a few studies yielded inconclusive results due to comparable proportions of biofilm producers and non-producers (Montoya-Hinojosa et al., 2023; Flores-Ibarra et al., 2026; Veschetti et al., 2026), or relative comparisons to *P. aeruginosa* PaO1 without defining whether lower biofilm production still qualified *Achromobacter* isolates as biofilm producers (Sahl et al., 2023; Acharya et al., 2026), biofilm formation was the predominant phenotype across all sources.

### 6.1 Biofilm formation in CF *Achromobacter* isolates

Most *Achromobacter* isolates included in the literature we reviewed originated from CF patients (n = 691, 35 studies), including 334 longitudinal isolates from serial sampling (Trancassini et al., 2014; Ridderberg et al., 2015; Nielsen et al., 2016, 2017; Filipic et al., 2017; Firmida et al., 2017; Veschetti et al., 2020, 2026; Khademi et al., 2021; Sandri et al., 2021, 2022; Sahl et al., 2025), and three co-isolates obtained from the same CF patient (Menetrey et al., 2020; Besse et al., 2025). Across these CF-related studies, biofilm formation was the predominant phenotype, reported in 26/35 studies while a minority (6/35) mainly described weak or absent biofilm-forming phenotypes (Veschetti et al., 2020; Damar-Çelik et al., 2021; Khademi et al., 2021; Sandri et al., 2021; Mataracı-Kara et al., 2025; Sahl et al., 2025).

#### 6.1.1 Assessment of biofilm production intensity in CF isolates using the crystal violet assay

A focus on the nine studies that included CF strains and applied CV staining with Stepanović classification (Stepanović et al., 2000, 2007) for comparative purpose showed that the proportion of non-biofilm-producing CF isolates ranged from 0% (Trancassini et al., 2014; Firmida et al., 2017; Menetrey et al., 2020) to 73% (40/55) (Khademi et al., 2021) (**Table 2**). Overall, approximately 38% of CF isolates were classified as non-producers (Filipic et al., 2017; Damar-Çelik et al., 2021; Khademi et al., 2021; Sandri et al., 2022; Mataracı-Kara et al., 2025; Veschetti et al., 2026) and 62% produced biofilm to varying degrees (Trancassini et al., 2014; Filipic et al., 2017; Firmida et al., 2017; Menetrey et al., 2020; Damar-Çelik et al., 2021; Sandri et al., 2022; Mataracı-Kara et al., 2025), acknowledging a probable overlap between two studies (Mataracı-Kara et al., 2025); (Damar-Çelik et al., 2021).

**Table 2:** Biofilm production of *Achromobacter* spp. by crystal violet assay (Stepanović classification): scoping review by origin.

Origin	Reference	No of strains	Non BP	Weak BP	Moderate BP	Strong BP
CF	*1*	52	–	7	16	29
	*2*	32	2	6	15	9
	*3*	28	–	4	–	24
	*4*	3	–	–	1	2
	*5^a^*	52	37	8	2	5
	*6*	95	23	44	22	6
	*7*	52	35	11	2	4
	Total (%)	314 (100)	97 (30.9)	80 (25.5)	58 (18.5)	79 (25.1)
	*8^b^*	55	40	15*^b^*		
	*9^b^*	22	11	11*^b^*		
Non-CF	*1*	5	–	–	–	5
	*2*	37	–	10	16	11
	*10*	15	–	5	6	4
	*11^c^*	1	–	–	–	1
	*12*	2	1	–	–	1
	*13^d^*	24	22	1	–	1
	*14^d^*	7	4	–	–	3
	Total (%)	91 (100)	27 (29.7)	16 (17.6)	22 (24.2)	26 (28.5)
ENV	*15^e^*	15	15*^e^*		–	–
	Total (%)	15 (100)	15 (100)		-	-
*A. xylosoxidans* type strain	*16*	1	–	–	–	1
	Total	1	-	-	-	1
OVERALL (%)		498 (100)	175 (≥35.1)*^f^*	96 (≥19.3)*^f,g^*	80 (≥16.1)*^g^*	106 (≥21.3)*^g^*

Percentages were calculated and reported in parentheses only when the total number of strains exceeded 10. Statistical analysis is presented in the main text.

*^a^*includes the *A. xylosoxidans* type strain, although its biofilm production category was not specified by the authors;

*^b^*biofilm production was reported as a binary outcome, with no classification into weak, moderate, or strong producers precluding inclusion of these studies in statistical analyses comparing biofilm-forming capacity by strain origin (Khademi et al., 2021; Veschetti et al., 2026): the values were highlighted in light gray to indicate that they cover all three categories;

*^c^*material-derived non-CF clinical isolate (isolated from soft contact lens);

*^d^*CF/non-CF status not reported by authors; isolates were classified as non-CF in this scoping review after examining their clinical origin;

*^e^*no distinction made between non-biofilm producers and weak biofilm producers precluding inclusion of this study in statistical analyses comparing biofilm-forming capacity by strain origin (Abdouchakour et al., 2015): the value “15” was highlighted in light gray to indicate that it covers both categories;

*^f^*excluding the 15 strains for which no distinction was made between non-biofilm producers and weak biofilm producers (Abdouchakour et al., 2015);

*^g^*excluding the biofilm-producing strains that were not classified into weak, moderate, or strong producers (Khademi et al., 2021; Veschetti et al., 2026). References are abbreviated as italicized numbers: *1* (Trancassini et al., 2014); *2* (Filipic et al., 2017); *3* (Firmida et al., 2017); *4* (Menetrey et al., 2020); *5* (Damar-Çelik et al., 2021); *6* (Sandri et al., 2022); *7* (Mataracı-Kara et al., 2025); *8* (Khademi et al., 2021); *9* (Veschetti et al., 2026); *10* (Liu et al., 2016); *11* (Konstantinović et al., 2017); *12* (Montoya-Hinojosa et al., 2023); *13* (Montoya-Hinojosa et al., 2024); *14* (Flores-Ibarra et al., 2026); *15* (Abdouchakour et al., 2015); *16* (Bellavita et al., 2023). A dash (-) indicates that no isolates were reported. BP, biofilm producer; CF, cystic fibrosis; ENV, environmental; No, number.

Conversely, the same methodological approaches also identified strong biofilm producers among CF isolates, with reported frequencies ranging from ~6% (6/95) (Sandri et al., 2022) to 86% (24/28) (Firmida et al., 2017), although the latter was influenced by two CF siblings colonized by clonally related strains. Taken together, around 25% of CF *Achromobacter* isolates were classified as strong producers (Trancassini et al., 2014; Filipic et al., 2017; Firmida et al., 2017; Menetrey et al., 2020; Damar-Çelik et al., 2021; Sandri et al., 2022; Mataracı-Kara et al., 2025), apart from the studies by Khademi et al. and Veschetti et al., which only used binary scoring (Khademi et al., 2021; Veschetti et al., 2026) (**Table 2**).

Comparative studies with *P. aeruginosa* PaO1 showed that CF *Achromobacter* isolates generally produced less biofilm. Jackobsen et al. showed that a CF *A. xylosoxidans* isolate adhered less than *P. aeruginosa* PaO1 after 48 h but still formed biofilm-like aggregates supported by the *pgaABCD* operon (Jakobsen et al., 2013). Similarly, Nielsen et al. reported reduced biofilm biomass in 19/23 CF *Achromobacter* isolates relative to *P. aeruginosa* PaO1 (Nielsen et al., 2016), a trend latter confirmed by Sahl et al. (Sahl et al., 2025). Consistent with these findings, Veschetti et al. reported poor polystyrene adhesion of four CF-derived *Achromobacter* strains using CV staining (Veschetti et al., 2020).

#### 6.1.2 Biofilm formation in CF *Achromobacter* isolates during persistent colonization

Longitudinal studies indicate that biofilm production in CF isolates may be stable or decline over time, depending on the strain and the stage at which the isolate was recovered during infection. Several studies document a gradual decrease in biofilm production over the course of chronic colonization, consistent with *Achromobacter*’s adaptation to the CF lung environment. In isolates collected 1–3 years apart, lower adhesion and biofilm production were associated with mutations in adhesion-related genes (*rmlA*, *rmlC*, *lapE*, O-antigen acetylase and diguanylate cyclase) (Ridderberg et al., 2015) or in the pathoadaptive repressor *bigR* (biofilm growth-associated repressor) (Khademi et al., 2021) (**Figure 3**). A cohort of 20 isolates, including 15 longitudinal strains from five CF patients similarly showed that early isolates produced more attached biofilm than later isolates collected approximately one to four years after the initial colonization, suggesting within-host adaptation (Nielsen et al., 2016). Conversely, other longitudinal series demonstrated stable biofilm phenotypes, with no significant differences over periods ranging from 469 days to six years (Veschetti et al., 2020; Sandri et al., 2022). This stability was also observed between paired sputum and nasal lavage isolates from the same CF patients, indicating that biofilm formation is largely conserved across airway niches within a given clone, despite occasional evidence of within-host diversification (Veschetti et al., 2026). Persistent high-level production was also documented in two CF siblings chronically colonized for more than ten years by clonally identical *A. xylosoxidans* isolates (Firmida et al., 2017). Besides, longitudinal data from a chronically colonized CF patient showed a progressive increase in tolerance of *A. xylosoxidans* biofilm to levofloxacin, colistin, piperacillin, and tobramycin over seven years (Nielsen et al., 2017), suggesting that biofilm-associated tolerance may evolve within-host persistence.

Importantly, biofilm-forming capacity did not differ between isolates from chronic *vs.* sporadic infections across three large studies including 52 strains from 34 CF patients (Trancassini et al., 2014), 122 strains from 39 CF patients (Pereira et al., 2017), and in a comparison of 16 isolates from 14 sporadically infected patients *vs.* 79 isolates from 24 chronically infected patients (Sandri et al., 2022).

Overall, CF-derived *Achromobacter* isolates frequently form biofilm, but their phenotypes show marked inter-strain variability, influenced both by the duration of colonization and within-host genetic adaptations. Taken together, these data highlight that biofilm formation is a widespread but heterogeneous trait among CF *Achromobacter* isolates.

### 6.2 Biofilm formation in non-CF clinical *Achromobacter* isolates

Biofilm formation was also studied in non-CF clinical contexts (n = 26 studies), encompassing 156 *Achromobacter* isolates from a wide range of clinical sources. Biofilm formation was the predominant phenotype, reported in 22 studies, whereas a single study mainly described a non-biofilm-forming phenotype (22/24 isolates) (Montoya-Hinojosa et al., 2024).

#### 6.2.1 Biofilm formation in material-derived clinical isolates

Although only four *Achromobacter* isolates from biological materials or medical devices have been reported to date (Kim et al., 2008; Konstantinović et al., 2017; Vijay and Willcox, 2018; Jean-Pierre et al., 2026) (**Table 3**), material-associated infections clearly illustrate the ability of these bacteria to persist in non-CF settings through biofilm formation.

**Table 3:** Origin of non-CF *Achromobacter* isolates according to their ability to form biofilm in the reviewed studies*^a^*.

Origin	Reference	Sample source	No of strains	Non-BP	BP	Difficult to interpret*^b^*
Material derived clinical non-CF isolates	*1,2*	catheter	2	–	2	–
	*3*	contact lens case	1	–	1	–
	*4*	soft contact lens	1	–	1	–
	Total material samples		4	-	4	-
Non-material derived clinical non-CF isolates	*5*	tracheal aspirate	25	–	25	–
	*6*	respiratory and sterile fluid samples	24	22	2	–
	*2,5,7,8*	sputum	18	–	11	7
	*2,5,8,9*	bronchoalveolar lavage	15	–	10	5
	*10*	hospital acquired pneumonia	15	–	15	–
	*2*	endobronchial aspirates	3	–	3	–
	*11*	NS respiratory tract specimen	3	–	3	–
	*2*	distal airway secretions	2	–	2	–
	*8*	autopsy lung specimen	2	–	–	2
	Total respiratory samples (%)		107 (100)	22 (20.6)	71 (66.3)	14 (13.1)
	*2*	NS ENT sites	5	–	5	–
	*5,8,12*	ear	3	–	3	–
	*13*	sinus	1	–	1	–
	*14*	buccal cavity	1	–	1	–
	*5*	nose	1	–	1	–
	Total ENT samples (%)		11 (100)	-	11 (100)	-
	*2,5,8,11,15,16*	blood culture	12	1	8	3
	*2,8,12,17,18*	skin and soft tissue	9	–	6	3
	*2,8*	rectum	2	–	1	1
	*2*	bone biopsy	1	–	1	–
	*2*	eye	1	–	1	–
	*8*	urinary	1	–	–	1
	*15*	peritoneal fluid	1	–	1	–
OVERALL (%)			149 (100)	23 (15.4)	104 (69.8)	22 (14.8)

Percentages were calculated and reported in parentheses only when the total number of strains exceeded 10.

*^a^*one study reporting seven non-CF isolates (three biofilm producers) was excluded from this analysis because the clinical origins of the isolates were not specified (Flores-Ibarra et al., 2026);

*^b^*inconclusive results because all *Achromobacter* isolates produced less biofilm than *P. aeruginosa* PaO1, yet no criteria were provided to determine whether they should still be classified as biofilm producers (Acharya et al., 2026). References are abbreviated as italicized numbers: *1* (Kim et al., 2008); *2* (Jean-Pierre et al., 2026); *3* (Vijay and Willcox, 2018); *4* (Konstantinović et al., 2017); *5* (Filipic et al., 2017); *6* (Montoya-Hinojosa et al., 2024); *7* (Al-Kahachi et al., 2023); *8* (Acharya et al., 2026); *9* (Flores-Maldonado et al., 2025); *10* (Liu et al., 2016); *11* (Trancassini et al., 2014); *12* (Marion-Sanchez et al., 2020); *13* (Hugon et al., 2015); *14* (De Souza Freitas et al., 2020); *15* (Montoya-Hinojosa et al., 2023); *16* (Vandeplassche et al., 2017); *17* (Li et al., 2013); *18* (Ulloa et al., 2020). A dash (-) indicates that no isolates were reported. BP, biofilm producer; CF, cystic fibrosis; ENT, ear, nose and throat; No, number; NS, non-specified.

On intravascular devices, *Achromobacter* was found embedded within a fibrin-like matrix on an explanted central venous catheter from an asymptomatic patient (Kim et al., 2008), and *in vivo*/clinical biofilm formation was documented on a prosthetic valve in an elderly patient with endocarditis (Wackernagel et al., 2025).

Biofilm has also been observed on both the internal and external surfaces of contact lenses in patients with keratitis (Wiley et al., 2012). Adhesion is material-dependent: it is lower on hydrogel lenses (etafilcon A), higher on silicone hydrogel materials (senofilcon A and comfilcon A), and further enhanced in the presence of organic soil (Vijay and Willcox, 2018). Additionally, Konstantinović et al. isolated a strong biofilm-producing *Achromobacter* sp. isolate from a soft contact lens (**Table 2**, **3**), further highlighting the potential of contact lenses to act as persistent reservoirs for *Achromobacter* biofilm that facilitate biofilm-associated eye infections (Konstantinović et al., 2017).

*Achromobacter* has been identified within biofilm from patients with catheter-associated urinary tract infections (CAUTIs), including following radical prostatectomy (Frank et al., 2009), in individuals with neurogenic bladder (Choe et al., 2012), and in an experimental rat model (Choe et al., 2013). One clinical study reported that 25% of urinary catheters (two out of eight) were colonized by *Achromobacter* sp., co-occurring with other bacteria (Frank et al., 2009).

Together, these observations confirm that bacteria of the *Achromobacter* genus can readily colonize and persist on a wide range of medical devices in non-CF contexts through robust biofilm formation, reinforcing its potential role in device-associated infections.

#### 6.2.2 Biofilm formation in non-material-derived clinical isolates

A total of 152 non-material clinical isolates were reported (Li et al., 2013; Trancassini et al., 2014; Hugon et al., 2015; Liu et al., 2016; Filipic et al., 2017; Vandeplassche et al., 2017; De Souza Freitas et al., 2020; Marion-Sanchez et al., 2020; Ulloa et al., 2020; Al-Kahachi et al., 2023; Montoya-Hinojosa et al., 2023, 2024; Flores-Maldonado et al., 2025; Acharya et al., 2026; Flores-Ibarra et al., 2026; Jean-Pierre et al., 2026), mostly from respiratory samples, reflecting the overall distribution of *Achromobacter* infections (Bayless et al., 2025) (**Table 3**).

Overall, all material derived clinical isolates and isolates from ear, nose, and throat samples were reported as biofilm producers. However, no clear association could be made between the site of isolation and biofilm-forming capacity, given the limited sample size of certain sources and the heterogeneity of assessment methods (**Table 3**).

#### 6.2.3 Assessment of biofilm production intensity in non-CF isolates using the crystal violet assay

Among the seven studies including non-CF strains and using the CV assay interpreted with the Stepanović criteria (Stepanović et al., 2000, 2007), 28.5% of *Achromobacter* isolates were classified as strong biofilm producers, whereas 29.7% were non-producers, illustrating substantial inter-strain variability (Trancassini et al., 2014; Liu et al., 2016; Filipic et al., 2017; Konstantinović et al., 2017; Montoya-Hinojosa et al., 2023, 2024; Flores-Ibarra et al., 2026) (**Table 2**). This heterogeneity is exemplified by two contrasting studies: Trancassini et al. reported that all five isolates examined were strong biofilm producers (Trancassini et al., 2014), whereas Montoya-Hinojosa et al. found that 92% of isolates (22/24) were non-producers (Montoya-Hinojosa et al., 2024). Finally, a comparative study with *P. aeruginosa* PaO1 found that only three of 26 *A. xylosoxidans* strains (11.5%) - originating from ear effusion, sputum, and bronchoalveolar lavage fluid samples - displayed substantial biofilm formation relative to PaO1, consistent with findings reported for CF isolates (Acharya et al., 2026).

### 6.3 Biofilm formation in environment-borne *Achromobacter* isolates

Biofilm formation was also studied in environmental contexts (n = 24 studies), encompassing 39 *Achromobacter* isolates from diverse healthcare and non-healthcare sources, reflecting the ecological adaptability of this genus. Biofilm formation was the predominant phenotype (87.5% of studies; 21/24), whereas the remaining three studies mainly reported a non-biofilm-forming phenotype (Abdouchakour et al., 2015; Yadav et al., 2023; Soon et al., 2025).

#### 6.3.1 Biofilm production in isolates from healthcare environments

Biofilm-producing *Achromobacter* spp. have been repeatedly isolated from disinfectant-associated reservoirs such as surface disinfectant solutions (n = 3) (Kampf et al., 2014; Hugon et al., 2015; Marion-Sanchez et al., 2020), tissue dispenser buckets containing disinfectant (Günther et al., 2016), and a sink drain exposed to disinfectant solutions (n = 1) (Marion-Sanchez et al., 2020). One outbreak underscored the clinical relevance of these findings: contaminated disinfectant atomizers were identified as the source of a pediatric onco-hematology bacteremia infection cluster (Hugon et al., 2015). Beyond disinfectant-related niches, *Achromobacter* sp. have also been detected in sink drain biofilm and hospital wastewater from intensive care units (Hennebique et al., 2024), highlighting their ability to persist in moist environments. In addition, *Achromobacter* has also been isolated from dental unit waterlines (n = 18) where biofilm phenotypes ranged from weak (Abdouchakour et al., 2015) to strong producers (Liaqat and Sabri, 2009; Liaqat et al., 2009, 2010), illustrating marked strain-dependent variability even within a single ecological reservoir.

#### 6.3.2 Biofilm production in isolates from non-healthcare environments

Biofilm-producing isolates have been documented in soil (Jakobsen et al., 2013; Li et al., 2013; Sato et al., 2026) and plant niches: rhizosphere (n = 1) (Kapadia et al., 2022) and mungbean roots (n = 1) (Zahra et al., 2023), but not in the aerial roots of *Rhynchostylis retusa* (n = 1) (Yadav et al., 2023).

Hydric environments, with varying levels of anthropization, represent another major reservoir in: domestic drains (n = 1) (McBain et al., 2003), sewage sludge (n = 2) (Azevedo et al., 2014, 2016), cooling water (n = 2) (Narenkumar et al., 2019), petroleum-contaminated seawater (n = 1) (Lazzem et al., 2023), and one unspecified water source (Nielsen et al., 2016). Moreover, *Achromobacter* sp. isolates have also been identified within biofilm formed on abiotic surfaces such as plastic carriers (Cao et al., 2022) and microplastics (Deng et al., 2024). However, biofilm capacity showed strong strain-dependence, with some isolates producing biofilm only under specific temperature or growth conditions (Lazzem et al., 2023), while one isolate recovered from a drinking water vending machine did not display a biofilm phenotype (Soon et al., 2025).

#### 6.3.3 Lack of longitudinal data on biofilm formation in environment-borne *Achromobacter* isolates

Only one environmental study included serial isolates, showing no clear temporal evolution in biofilm formation, mirroring observations from some longitudinal CF cohorts (Abdouchakour et al., 2015).

### 6.4 Statistical analyses comparing biofilm formation according to origin of isolates

Overall, limited to CV-based studies applying the Stepanović classification for comparative purposes, the capacity to produce biofilm did not differ significantly between CF and non-CF isolates (χ^2^ test, *p* = 0.1438), with 62.1% of CF isolates (243/391) and 70.3% of non-CF isolates (64/91) being biofilm producers (**Table 2**). Differences in intensity (weak and moderate *vs.* strong producers) were also non-significant (χ^2^ test, *p* = 0.5398). The only study meeting these criteria and including environmental isolates could not be included in the statistical analysis due to the lack of distinction between non- and weak biofilm producers (Abdouchakour et al., 2015). The limited environmental data precluded statistical comparison with clinical isolates, highlighting a gap in the literature and the need for further studies on environment-borne isolates.

Thus, across CF and non-CF settings, the current evidence does not support any consistent association between the origin of isolation and the ability of *Achromobacter* to form biofilm. Instead, biofilm phenotypes exhibit marked strain-to-strain variability, consistent with Acharya et al. (Acharya et al., 2026). Although CF isolates demonstrate stronger binding to host-associated substrates such as mucin, collagen, and fibronectin (Filipic et al., 2017), and our recent work shows faster early adhesion in non-CF isolates (Jean-Pierre et al., 2026), their overall biofilm-forming capacity does not differ between CF and non-CF isolates. These findings support a strain-dependent, rather than CF/non-CF origin-dependent biofilm phenotype in *Achromobacter*.

## 7 Effects of interaction between *Achromobacter* and other microorganisms on biofilm formation

A total of 12 studies investigated multi-species biofilm involving *Achromobacter* sp., most often in dual-species settings. These works reveal complex, strain-dependent interactions shaping biofilm architecture, bacterial competition, and antibiotic tolerance (Frank et al., 2009; Azevedo et al., 2014, 2016; Abdouchakour et al., 2015; Vandeplassche et al., 2017, 2020; Menetrey et al., 2020; Sandri et al., 2021; Bellavita et al., 2023; Yilmaz et al., 2023; Besse et al., 2025; Sahl et al., 2025).

### 7.1 Influence of *Achromobacter* on cocultured microorganisms’ biofilm

#### 7.1.1 Influence of *Achromobacter* on *P. aeruginosa* biofilm

Because *Achromobacter* sp. and *P. aeruginosa* are frequently co-isolated in CF airways, their interplay has been widely studied, revealing three distinct interaction patterns: enhancement of *P. aeruginosa* biofilm (Abdouchakour et al., 2015), reduction of biofilm formation (Menetrey et al., 2020; Sandri et al., 2021; Besse et al., 2025; Sahl et al., 2025), or no detectable effect (Sandri et al., 2021; Yilmaz et al., 2023; Sahl et al., 2025) (**Figure 5**).

**Figure 5:**
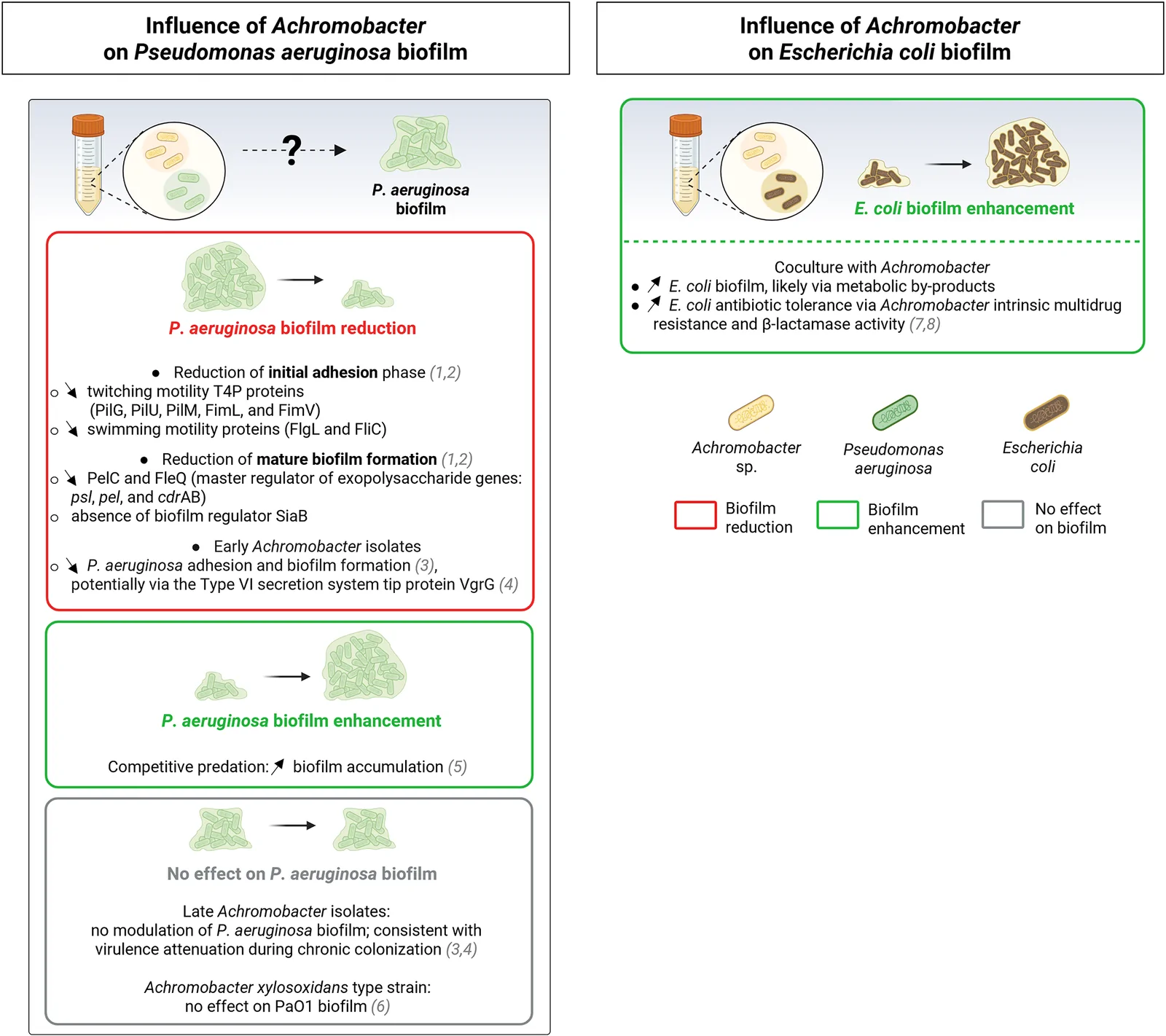
Influence of *Achromobacter* on *Pseudomonas aeruginosa* and *Escherichia coli* biofilm. The references are indicated by italicized numbers in parentheses: *1* (Menetrey et al., 2020); *2* (Besse et al., 2025); *3* (Sahl et al., 2025); *4* (Sandri et al., 2021); *5* (Abdouchakour et al., 2015); *6* (Yilmaz et al., 2023); *7* (Azevedo et al., 2014); *8* (Azevedo et al., 2016).

##### 7.1.1.1 Enhancement of *P. aeruginosa* biofilm

One environmental study using the CV assay reported greater *P. aeruginosa* biofilm biomass when cocultured with *Achromobacter* sp (Abdouchakour et al., 2015). However, *Achromobacter* disappeared from the mature biofilm, indicating competitive predation by *P. aeruginosa* rather than synergistic cooperation (Abdouchakour et al., 2015) (**Figure 5**).

##### 7.1.1.2 Reduction of *P. aeruginosa* biofilm

Coculture of six *A. xylosoxidans*/*P. aeruginosa* clinical pairs, involving three *A. xylosoxidans* strains (*Ax* 198, *Ax* 199, *Ax* 200), three *P. aeruginosa* strains (*Pa* I.14, *Pa* I.31, *Pa* II.17), plus one environmental strain (*Pa* 50) from the same CF patient, reduced biofilm formation for *P. aeruginosa* and/or *A. xylosoxidans* compared with monocultures, using the CV assay. These effects were largely independent of growth inhibition, except for two combinations (*Pa* II.17 with *Ax* 199 or *Ax* 200) (Menetrey et al., 2020). Recent proteomic analyses (Besse et al., 2025) have confirmed these findings (Menetrey et al., 2020). Coculture with two *A. xylosoxidans*/*P. aeruginosa* pairs (*Ax* 198/*Pa* II.17 and *Ax* 200/*Pa* II.17) caused a broad reduction in *P. aeruginosa* proteins essential for biofilm formation. In the early adhesion phase, twitching motility was impaired due to decreased abundance of Type IV pili components (PilG, PilU, PilM, FimL, FimV), and swimming motility proteins (FlgL, FliC) were also reduced, probably affecting surface attachment. In the later stages, EPS synthesis and biofilm matrix formation were compromised, as evidenced by reduced PelC and FleQ (the master regulator of biofilm formation that activates EPS-related genes *psl*, *pel*, and *cdr*AB), and the biofilm regulator SiaB was absent. Collectively, these proteomic changes support a strong inhibitory effect of *Ax* 200 on *Pa* II.17 biofilm formation (**Figure 5**). In contrast, coculture with *Ax* 198 had milder proteomic effects on *Pa* II.17 biofilm-associated proteome, yet the phenotype with CV-based assays still showed a reduction in biofilm formation (Besse et al., 2025).

In coculture experiments using early and late *A. xylosoxidans*/*P. aeruginosa* clinical pairs collected longitudinally from two CF patients chronically co-colonized by both species, only early *Achromobacter* isolates reduced *P. aeruginosa* biofilm (Sandri et al., 2021). Moreover, a competitive *A. insuavis* isolate inhibited *P. aeruginosa* adhesion (Sandri et al., 2021). Genomic analysis identified the presence of the VgrG Type VI secretion system tip protein as a key factor known to mediate inter-species competition and whose paralogs have also been involved in biofilm regulation (Sha et al., 2013) (**Figure 3**). These observations support strain- and stage-specific competitive behavior, not restricted to *A. xylosoxidans* (Menetrey et al., 2020; Besse et al., 2025).

Using six *A. xylosoxidans*/*P. aeruginosa* coculture pairs obtained from three CF patients chronically colonized by *Achromobacter* - including paired early and late *Achromobacter* isolates from each patient - and a single CF-derived *P. aeruginosa* strain, Sahl et al. showed that only early *A. xylosoxidans* isolates significantly reduced *P. aeruginosa* biofilm biomass. Biofilm inhibition occurred exclusively during the early stages of biofilm development, requiring live bacteria, depending on *A. xylosoxidans* concentration, and correlating with superior surface adhesion of early *A. xylosoxidans* isolates, which hindered *P. aeruginosa* attachment and subsequent biofilm establishment (Sahl et al., 2025).

##### 7.1.1.3 No detectable effect on *P. aeruginosa* biofilm

Several studies reported neutral interactions, especially with late-stage *Achromobacter* isolates. Late *A. xylosoxidans* and *A. insuavis* isolates had no effect on *P. aeruginosa* biofilm (Sandri et al., 2021; Sahl et al., 2025). Similarly, *A. xylosoxidans* type strain had no effect on *P. aeruginosa* PaO1 biofilm formation (Yilmaz et al., 2023). Together, these observations are in line with the concept of virulence attenuation during chronic CF adaptation (Menetrey et al., 2021), as *Achromobacter* shifts from competition with *P. aeruginosa* to tolerant cohabitation with it in polymicrobial biofilm.

#### 7.1.2 Influence of *Achromobacter* on *Escherichia coli* biofilm

*Achromobacter* can also interact with bacterial species other than *P. aeruginosa*, particularly in polymicrobial CAUTIs (Frank et al., 2009). Coculture with *Achromobacter* sp. enhanced *E. coli* biofilm formation, driven by *Achromobacter* metabolic by-products which promote *E. coli* adhesion and growth, conferring a competitive advantage in dual-species biofilm (Azevedo et al., 2014) (**Figure 5**). Moreover, this coculture led to well-organized microbial consortia with greater antibiotic tolerance. Under antibiotic exposure, *Achromobacter* sp. appears to protect *E. coli* with β-lactamase production by *Achromobacter*-hydrolyzed antibiotics inadvertently protecting *E. coli*, which benefits without metabolic cost, gaining a competitive advantage within the biofilm (Azevedo et al., 2016).

### 7.2 Influence of cocultured microorganisms on *Achromobacter* biofilm

The influence of cocultured microorganisms on *Achromobacter* biofilm formation was also investigated in seven studies (Azevedo et al., 2014; Abdouchakour et al., 2015; Vandeplassche et al., 2017, 2020; Sandri et al., 2021; Bellavita et al., 2023; Yilmaz et al., 2023), revealing three distinct outcomes (**Figure 6**).

**Figure 6:**
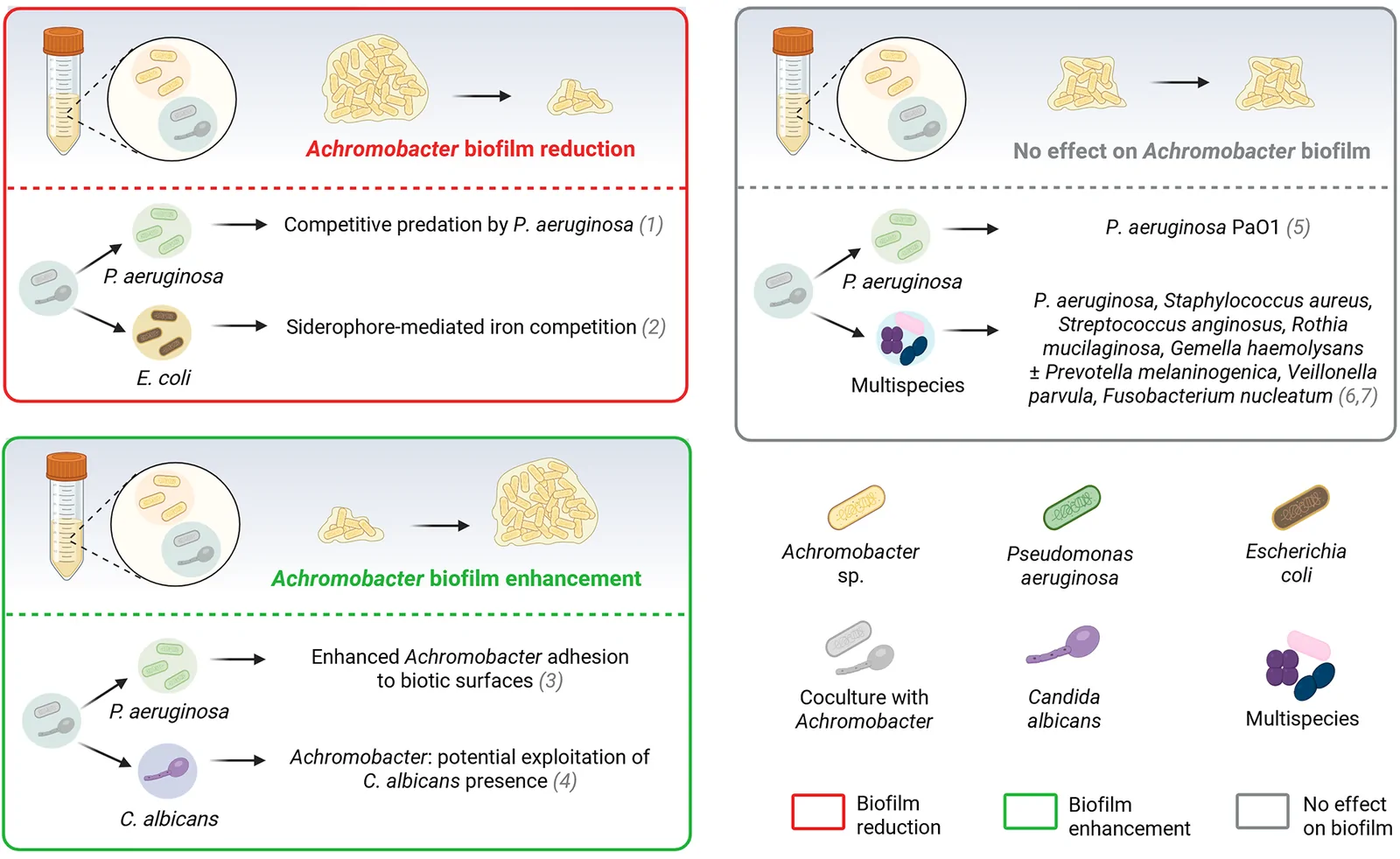
Influence of cocultured microorganisms on *Achromobacter* biofilm. The references are indicated by italicized numbers in parentheses: *1* (Abdouchakour et al., 2015); *2* (Azevedo et al., 2014); *3* (Sandri et al., 2021); *4* (Bellavita et al., 2023); *5* (Yilmaz et al., 2023); *6* (Vandeplassche et al., 2020); *7* (Vandeplassche et al., 2017). **Figure 3**-**6** were created with the assistance of BioRender.com (BioRender, Toronto, Canada). Numbers in brackets indicate the corresponding reference numbers.

#### 7.2.1 Reduction of *Achromobacter* biofilm

Two studies documented a decrease in *Achromobacter* biofilm (Azevedo et al., 2014; Abdouchakour et al., 2015) (**Figure 6**). Coculture with *E. coli* reduced *Achromobacter* biofilm formation, with *Achromobacter* sp. progressively outcompeted at later stages, probably due to the higher siderophore production of *E. coli* in iron-limited environments such as urine during CAUTIs (Azevedo et al., 2014). Similarly, in coculture with *P. aeruginosa*, *Achromobacter* sp. disappeared from mature biofilm, highlighting *P. aeruginosa*’s dominance in structured communities (Abdouchakour et al., 2015).

#### 7.2.2 Enhancement of *Achromobacter* biofilm

In CF dual infections, *P. aeruginosa* increased *Achromobacter*’s adhesion to biotic surfaces, promoting persistence within polymicrobial biofilm (Sandri et al., 2021). Moreover, in dual-species biofilm with *Candida albicans*, total biomass exceeded that of both mono-species biofilm, and *A. xylosoxidans* became progressively dominant, representing 55% of the biomass at 24 h and 64% at 48 h (Bellavita et al., 2023). These findings suggest that fungal presence may facilitate the creation of *A. xylosoxidans* biofilm and its persistence during CF lung colonization (**Figure 6**).

#### 7.2.3 No effect on *Achromobacter* biofilm

Two studies reported neutral effects on *Achromobacter* biofilm formation. In a coculture of the *A. xylosoxidans* type strain with *P. aeruginosa* PaO1, no change in *Achromobacter* biofilm was observed, except under amikacin exposure, which reduced *A. xylosoxidans* biofilm only in dual-species conditions (Yilmaz et al., 2023). In a multispecies microaerophilic biofilm model including *P. aeruginosa*, *Staphylococcus aureus*, *Streptococcus anginosus*, *Rothia mucilaginosa*, and *Gemella haemolysans*, *A. denitrificans* formed biofilm of similar density to its single-species condition, with no alteration in antibiotic susceptibility (Vandeplassche et al., 2017, 2020). Adding anaerobes such as *Prevotella melaninogenica*, *Veillonella parvula*, and *Fusobacterium nucleatum* had no effect on *A. denitrificans* biofilm formation or antibiotic tolerance either (Vandeplassche et al., 2020) (**Figure 6**).

In conclusion, interactions involving *Achromobacter* are highly strain-dependent, not species-driven. With *P. aeruginosa*, the relationship ranges from competition to neutral coexistence and occasional enhancement. Interaction outcomes often depend on isolate stage (early *vs.* late colonizers), ecological context (CF *vs.* CAUTI), metabolic cross-feeding, and interference competition (e.g., T6SS VgrG). In mixed communities, *Achromobacter* may either suppress competitors, facilitate their survival, or benefit from microbial partners (e.g., *Candida* scaffolding). These reciprocal interactions are essential for understanding *Achromobacter*’s persistence, antibiotic tolerance, and ecological plasticity.

## 8 Antibiofilm strategies targeting *Achromobacter* sp.

### 8.1 Antibiotics evaluated for antibiofilm activity

Over 18 studies using various methodological approaches (**Figure 4**), the *in vitro* antibiofilm activity of 20 antibiotics was assessed against 103 *Achromobacter* isolates, with four antibiotics also tested in combination with ceftazidime-avibactam (CTV). Approaches included microbiological methods (Liaqat et al., 2009; Azevedo et al., 2016; Nielsen et al., 2016, 2017; Tom et al., 2016; Vandeplassche et al., 2017, 2020; Ulloa et al., 2020; Aiyer et al., 2021; Yilmaz et al., 2023; Montoya-Hinojosa et al., 2024; Ebaid et al., 2025; Flores-Ibarra et al., 2026), chemical assays (Liaqat et al., 2009; Nielsen et al., 2016; Ulloa et al., 2020; Aiyer et al., 2022; Ebaid et al., 2025; Mataracı-Kara et al., 2025), and microscopy-based techniques (Azevedo et al., 2016; Tom et al., 2016; Cameron et al., 2019; Damar-Çelik et al., 2021; Yilmaz et al., 2023; Flores-Ibarra et al., 2026; Jean-Pierre et al., 2026). The study by Tom et al. represented the largest dataset (n = 68 isolates) (Tom et al., 2016), whereas all the other studies assessed a maximum of five isolates (median: 1.5). Two studies assessed anti-adhesion activity together with inhibition of biofilm formation (Damar-Çelik et al., 2021; Mataracı-Kara et al., 2025), whereas two others focused solely on the prevention of biofilm formation (Liaqat et al., 2009; Ebaid et al., 2025). In contrast, most studies (n = 13) investigated antibiotic activity exclusively on mature biofilm (Azevedo et al., 2016; Nielsen et al., 2016, 2017; Tom et al., 2016; Vandeplassche et al., 2017, 2020; Cameron et al., 2019; Ulloa et al., 2020; Aiyer et al., 2021, 2022; Yilmaz et al., 2023; Montoya-Hinojosa et al., 2024; Flores-Ibarra et al., 2026). Our recent study was the only one to assess both the reduction in biofilm formation and activity against mature biofilm in the same strains, under controlled microfluidic conditions (Jean-Pierre et al., 2026). The summary of *in vitro* antibiofilm activity is provided in **Table 4** according to the stage of biofilm development, with molecules demonstrating antibiofilm activity at each stage highlighted in green.

**Table 4:** *In vitro* antibiofilm activity of antibiotics and non-antibiotic compounds in *Achromobacter* spp. according to the stage of biofilm development.

Antibiofilm compounds	Adhesion		Biofilm formation		Mature biofilm		
Antibiotics							
Amikacin*	**-**		**-**		**2** *(6,7)*		
Amoxicillin-clavulanic acid	**-**		**-**		**1** *(8)*		
Ampicillin*	**-**		**-**		**1** *(8)*		
Azithromycin*	**-**		**-**		**1** *(9)*		
Aztreonam*	**-**		**-**		**2** *(6,7)*		
Ceftazidime	**-**		**-**		**1** *(10)*	**2** *(11,12)*	**2** *(13,14)*
Ceftazidime-avibactam (CTV)	**1** *(1)*		**1** *(1)*		**-**		
**Cefiderocol**	**-**		**1** *(3)*		**1** *(3)*		
Chloramphenicol	**1** *(1)*		**2** *(1,4)*		**-**		
Chloramphenicol + CTV	**1** *(1)*		**1** *(1)*		**-**		
Ciprofloxacin	**1** *(1)*		**2** *(1,5)*		**3** *(6,15,16)*	**2** *(8,13)*	**1** *(10)*
Ciprofloxacin + CTV	**1** *(1)*		**1** *(1)*		**-**		
Colistin*	**1** *(1)*		**1** *(1)*		**3** *(11,15,16)*	**2** *(7,10)*	**2** *(13,17)*
Colistin* + CTV	**1** *(1)*		**1** *(1)*		**-**		
Gentamicin*	**-**		**-**		**1** *(8)*		
**Imipenem**	**-**		**1** *(3)*		**1** *(3)*		
Levofloxacin	**-**		**-**		**1** *(18)*	**2** *(7,17)*	
**Meropenem**	**1** *(2)*	**1** *(1)*	**2** *(1,2)*	**1** *(3)*	**2** *(3,12)*	**1** *(14)*	
Meropenem + CTV	**1** *(1)*		**1** *(1)*		**-**		
Piperacillin	**-**		**-**		**1** *(17)*		
**Piperacillin-tazobactam**	**-**		**1** *(3)*		**1** *(3)*	**1** *(11)*	
Tobramycin*	**-**		**-**		**3** *(7,13,17)*	**2** *(6,18)*	**2** *(10,11)*
**Trimethoprim-sulfamethoxazole**	**-**		**1** *(3)*		**1** *(3)*	**1** *(12)*	**1** *(14)*
Tetracycline*	**-**		**1** *(4)*		**-**		
Disinfectants							
Benzalkonium chloride and polyaminopropyl biguanide	**-**		**-**		**1** *(26)*		
Didecyl dimethyl ammonium chloride	**-**		**-**		**1** *(27)*		
Mikrobac forte, Terralin protect, and Incidin plus	**-**		**1** *(19)*		**-**		
Povidone-iodine contact lens solution	**-**		**-**		**1** *(28)*		
Triclosan	**-**		**1** *(20)*		**-**		
Others							
Bacteriophages (Ade2 and Axy4)					**1** *(14)*		
Ceragenins (CSA-13, CSA-131, and CSA-131-P)	**1** *(2)*		**1** *(2)*		**1** *(2)*		
Coumarin-thiazole hybrids (6a and 6c)	**-**		**1** *(5)*		**-**		
Curcumin-chitosan-sodium tripolyphosphate magnetic nanoparticules	**-**		**1** *(21)*		**1** *(21)*		
DNase I, subtilisin, and alginate lyase	**-**		**-**		**1** *(11)*		
Efflux pump inhibitor phenylalanine-arginyl-β-naphthylamide	**1** *(17)*		**-**		**-**		
Fengycins and iturins	**-**		**-**		**1** *(29)*		
Gallic acid	**-**		**1** *(22)*		**-**		
*Lactobacillus plantarum* supernatants	**-**		**1** *(23)*		**1** *(23)*		
Methyl gallate	**-**		**1** *(22)*		**-**		
N-acetylcysteine	**-**		**-**		**3** *(6,15,16)*		
NB-401 nanoemulsion	**-**		**-**		**1** *(30)*		
Patulin and penicillic acid ± EDTA	**-**		**1** *(24)*		**-**		
WMR-4	**-**		**1** *(25)*		**1** *(25)*		

Antibiotics and non-antibiotic compounds are listed alphabetically and categorized according to their *in vitro* effects reported on three stages of biofilm development in *Achromobacter* spp.: Adhesion (Column 1), Biofilm formation (Column 2), and Mature biofilm (Column 3). Cell colors indicate the reported effect: green = antibiofilm activity; gray = no effect; red = biofilm enhancement; and blue = effect difficult to interpret based on the available data, as these agents are reported to be less effective than others without clarification on whether they retain any activity. Antibiotics representing treatment options against *Achromobacter* infections are highlighted in bold (Isler et al., 2020; Bayless et al., 2025). An asterisk (*) denotes antibiotics to which *Achromobacter* spp. are expected to exhibit a resistant phenotype. Numbers in bold indicate the number of studies reporting the corresponding effect, with the associated references indicated by italicized numbers in parentheses: *1* (Mataracı-Kara et al., 2025); *2* (Damar-Çelik et al., 2021); *3* (Jean-Pierre et al., 2026); *4* (Liaqat et al., 2009); *5* (Ebaid et al., 2025); *6* (Yilmaz et al., 2023); *7* (Tom et al., 2016); *8* (Azevedo et al., 2016); *9* (Ulloa et al., 2020); *10* (Vandeplassche et al., 2020); *11* (Nielsen et al., 2016); *12* (Montoya-Hinojosa et al., 2024); *13* (Vandeplassche et al., 2017); *14* (Flores-Ibarra et al., 2026); *15* (Aiyer et al., 2021); *16* (Aiyer et al., 2022); *17* (Nielsen et al., 2017); *18* (Cameron et al., 2019); *19* (Kampf et al., 2014); *20* (McBain et al., 2003); *21* (Montoya-Hinojosa et al., 2023); *22* (Flores-Maldonado et al., 2025); *23* (Danilova et al., 2022); *24* (Liaqat et al., 2010); *25* (Bellavita et al., 2023); *26* (Chang et al., 2015); *27* (Hugon et al., 2015); *28* (Yamasaki et al., 2018); *29* (De Souza Freitas et al., 2020); *30* (LiPuma et al., 2009). A dash (-) indicates that no data were available.

#### 8.1.1 *In vitro* antibiofilm effects of antibiotics associated with an expected resistant phenotype in *Achromobacter*

Although *A. xylosoxidans* is expected to be resistant to several antibiotic classes, some of these agents have nonetheless been investigated for potential antibiofilm activity.

Colistin was the most commonly studied agent in the literature on *Achromobacter* antibiofilm. Among eight studies, colistin showed activity against mature biofilm (Nielsen et al., 2016; Aiyer et al., 2021, 2022), inhibition of biofilm formation (Mataracı-Kara et al., 2025), and greater efficacy against biofilm when combined with CTV, including inhibition of early adhesion (Mataracı-Kara et al., 2025). Aminoglycosides, including tobramycin, amikacin, and gentamicin, have also been evaluated against mature *Achromobacter* biofilm. Among them, tobramycin was the only agent to show antibiofilm activity (Cameron et al., 2019; Yilmaz et al., 2023), reported in two out of seven studies, underscoring the limited, inconsistent efficacy of this antibiotic class. Azithromycin, the only macrolide evaluated, demonstrated activity against mature biofilm in a single study (Ulloa et al., 2020). Tetracycline also inhibited biofilm formation in one study (Liaqat et al., 2009). Finally, β-lactams to which *Achromobacter* is intrinsically resistant - more specifically, ampicillin (Azevedo et al., 2016) and aztreonam (Tom et al., 2016; Yilmaz et al., 2023) - showed no activity against mature biofilm (**Table 4**).

#### 8.1.2 Variable effects of clinically relevant antibiotics on *Achromobacter* biofilm

##### 8.1.2.1 *In vitro* antibiofilm activity

Recent susceptibility data support the use of trimethoprim-sulfamethoxazole, piperacillin-tazobactam, and the two carbapenems meropenem and imipenem as first-line treatment options for *Achromobacter* infections (Isler et al., 2020; Bayless et al., 2025). However, their antibiofilm performance has been inconsistently evaluated across studies (**Table 4**).

The antibiofilm activity of trimethoprim-sulfamethoxazole was assessed in three studies with inconsistent results. One study, conducted on two clinical isolates with unreported CF status, reported minimum biofilm eradication concentration (MBEC) values, defined as the lowest concentration of an antimicrobial agent required to eradicate a preformed biofilm, far exceeding MICs, suggesting poor activity against mature biofilm (Montoya-Hinojosa et al., 2024). In contrast, our recent work demonstrated consistent reduction of mature biofilm in four strains, either from CF or non-CF patients (Jean-Pierre et al., 2026). These observations indicate that trimethoprim-sulfamethoxazole’s efficacy against biofilm is strain-dependent. Similarly, the evidence regarding piperacillin-tazobactam also shows inconsistent, often limited, antibiofilm activity. In our experiments, sub-inhibitory concentrations enhanced biofilm formation in all five strains tested, although piperacillin-tazobactam produced modest reductions in mature biofilm (Jean-Pierre et al., 2026). Conversely, another study reported very high MBEC values, indicating that piperacillin-tazobactam may have limited benefits against established *Achromobacter* biofilm (Nielsen et al., 2016). Among carbapenems, meropenem exhibits some of the most contradictory results. Depending on the study, meropenem either inhibited adhesion and biofilm formation (Damar-Çelik et al., 2021) or reduced biofilm formation without affecting adhesion (Mataracı-Kara et al., 2025). Its combination with CTV maintained antibiofilm activity and significantly enhanced anti-adhesion effects (Mataracı-Kara et al., 2025). However, in our study, sub-minimal inhibitory concentration (MIC) of meropenem increased biofilm formation in all strains tested (Jean-Pierre et al., 2026). Despite these discrepancies, meropenem still shows activity against mature biofilm in certain isolates (Montoya-Hinojosa et al., 2024; Jean-Pierre et al., 2026). Similar trends were observed for imipenem; sub-MIC exposure enhanced biofilm formation, whereas imipenem displayed modest activity against mature biofilm (Jean-Pierre et al., 2026).

When resistance to first-line antibiotics is encountered, cefiderocol, a siderophore cephalosporin, is recommended (Isler et al., 2020). Cefiderocol exhibits high activity against planktonic *Achromobacter* (Jean-Pierre et al., 2024), and also showed potent *in vitro* antibiofilm activity, reducing both early biofilm formation at sub-MIC and preformed biofilm across all strains tested (Jean-Pierre et al., 2026) (**Table 4**).

Several other antibiotic classes, despite showing lower *in vitro* activity against planktonic *Achromobacter* isolates (Bayless et al., 2025), have also been evaluated for their antibiofilm properties (**Table 4**). Among β-lactams, ceftazidime demonstrates inconsistent activity (active against mature biofilm in 1/5 studies) (Vandeplassche et al., 2020). Similarly, piperacillin (Nielsen et al., 2017) and amoxicillin-clavulanate (Azevedo et al., 2016) are both ineffective against mature *Achromobacter* biofilm, as reflected by very high MBEC values. In contrast, fluoroquinolones appear to be among the most consistently active antibiotic class. Ciprofloxacin inhibits biofilm formation (Ebaid et al., 2025; Mataracı-Kara et al., 2025), reduces bacterial adhesion when combined with CTV (Mataracı-Kara et al., 2025), and is active against mature biofilm (Aiyer et al., 2021, 2022; Yilmaz et al., 2023). Levofloxacin also showed activity against mature biofilm in one study (Cameron et al., 2019). Collectively, these findings suggest that fluoroquinolones show promising, reproducible antibiofilm effects. Finally, chloramphenicol reduces *Achromobacter* biofilm formation alone or with CTV (Liaqat et al., 2009; Mataracı-Kara et al., 2025).

##### 8.1.2.2 Clinical evidence and therapeutic implications

Clinical evidence has also been reported. Two recent case reports support experimental findings. Trimethoprim-sulfamethoxazole was successful in a chronic scalp infection (Wan et al., 2025), consistent with the findings of our recent study demonstrating its *in vitro* activity against mature *Achromobacter* biofilm (Jean-Pierre et al., 2026). Using meropenem in combination with cefiderocol successfully treated prosthetic valve endocarditis following surgical valve replacement with coronary bypass (Wackernagel et al., 2025).

Overall, the antibiofilm activity of both trimethoprim-sulfamethoxazole and cefiderocol against *Achromobacter* spp. is supported by *in vitro* evidence demonstrating activity against both biofilm formation and mature biofilms, as well as by clinical observations, whereas *in vitro* data suggest that carbapenems and piperacillin-tazobactam are less effective and may promote biofilm formation at sub-inhibitory concentrations.

### 8.2 Non-antibiotic antibiofilm strategies

In the context of increasing antibiotic resistance and the challenge to treat biofilm-associated infections, growing attention has shifted toward non-antibiotic strategies to control *Achromobacter* biofilm. To date, 20 studies have explored such approaches, which can be broadly categorized into strategies targeting environmental reservoirs and those aimed at clinical infections (**Table 4**).

#### 8.2.1 Strategies targeting environmental *Achromobacter* biofilm

In environmental settings, disinfectants and antiseptics are widely used to prevent biofilm formation or eradicate established biofilm. However, several studies indicate that exposure to biocides may also select tolerant *Achromobacter* populations.

Triclosan (TCS; 2,4,4′-trichloro-2′-hydroxydiphenyl ether), a commonly used antiseptic since the 1960s, has been shown to promote the persistence of triclosan-tolerant bacteria within multispecies biofilm, including *Achromobacter* (McBain et al., 2003). Consistently, long-term exposure experiments to disinfectants (Mikrobac^®^ forte, Terralin^®^ Protect, Incidin™ Plus) increased *Achromobacter* sp. biofilm formation up to five times after 23 days of incubation (Kampf et al., 2014).

The activity of quaternary ammonium compounds, *i.e.*, didecyl dimethyl ammonium chloride (Surfanios^®^, 0.25%), has also been investigated. When 24-h preformed biofilm from two *Achromobacter* sp. strains were exposed for 4 h, only isolates from disinfectant sprays displayed partial survival (~25%), whereas a clinical sinusitis strain was completely eradicated (Hugon et al., 2015).

In the specific context of contact lens disinfection, *A. xylosoxidans* biofilm was more tolerant toward benzalkonium chloride and polyaminopropyl biguanide than *P. aeruginosa* biofilm, although benzalkonium chloride induced more pronounced membrane damage than polyaminopropyl biguanide at ophthalmic-use concentrations (Chang et al., 2015). In contrast, povidone-iodine-based contact lens care systems (Cleadew^®^, Ophtecs Corporation, Japan) were effective against mature biofilm formed by one *Achromobacter* sp. strain (Yamasaki et al., 2018) (**Table 4**).

Altogether, these different observations underlined *Achromobacter*’s strong overall capacity to adapt to chemical biocides, as well as its great facility to survive within biofilm.

#### 8.2.2 Strategies targeting clinical *Achromobacter* biofilm

##### 8.2.2.1 N-acetylcysteine

Despite its mechanistically plausible properties (matrix disruption, redox modulation, metal chelation) (Blasi et al., 2016), N-acetylcysteine (NAC) has shown minimal, inconsistent activity against *Achromobacter* biofilm. Treatments combining NAC with ciprofloxacin or colistin did not consistently reduce viable cell counts compared with antibiotic monotherapy, and neither synergistic nor additive effects were observed on mature biofilm. Moreover, NAC-associated effects varied markedly depending on the strain tested and the concentrations used, complicating interpretation of its therapeutic contribution (Aiyer et al., 2021, 2022). Similarly, Yilmaz et al. reported no dose-dependent effect of NAC on single- or dual-species biofilm involving *A. xylosoxidans* and *P. aeruginosa*. In that study, NAC alone exhibited minimal antibiofilm activity, and most antibiotics - including tobramycin, amikacin, and ciprofloxacin, with the exception of aztreonam - were less effective when combined with NAC than when used alone, possibly due to chemical interference impairing antibiotic activity (Landini et al., 2016; Yilmaz et al., 2023). Overall, current evidence indicates that NAC lacks reliable activity against *Achromobacter* biofilm (**Table 4**).

##### 8.2.2.2 Antimicrobial peptides and derivatives

Among alternative therapeutics, antimicrobial peptides have gained attention for their activity against multidrug-resistant pathogens, as well as their anti-inflammatory and immunomodulatory properties (Bellavita et al., 2023). An antimicrobial peptide derived from the marine peptide Myxinidin, named WMR, was optimized into a more serum-stable form, WMR-4. WMR-4 inhibited both biofilm formation and preformed biofilm in a concentration-dependent way, achieving near-complete inhibition of mono-species *A. xylosoxidans* biofilm and approximately 75% inhibition in mixed *C. albicans*-*A. xylosoxidans* biofilm at 20 µM. It partially disrupted preformed biofilm, reducing mono-species biofilm by 60% and dual-species biofilm by 40% at the same concentration. Gene expression analyses revealed downregulation of biofilm- and quorum-sensing-related genes (*epsF*, *AxyA*) and upregulation of the stress protein-encoding *uspA* gene (**Figure 3**). Biophysical studies showed strong interactions between WMR-4 and lipopolysaccharide and insertion into liposomes mimicking Gram-negative and *Candida* membranes, clarifying its mode of action (Bellavita et al., 2023). Overall, these results highlight WMR-4 as a promising therapeutic antimicrobial peptide for managing *Achromobacter* biofilm in chronic infections, including those in CF patients (Bellavita et al., 2023) (**Table 4**).

Ceragenins, or cationic steroid antibiotics (CSAs), are bile acid-derived amphiphilic compounds designed to mimic the antibacterial activity of natural AMPs. By targeting microbial membranes through electrostatic interactions, ceragenins induce transient pore formation, membrane depolarization, and cell death. Unlike AMPs, they are resistant to proteolysis and can be synthesized on a large scale, offering practical advantages for therapeutic applications (Damar-Çelik et al., 2021). The antibiofilm activities of three ceragenins (CSA-13, CSA-131, and CSA-131-P) were evaluated against five strong biofilm-producing *Achromobacter* sp. isolates using the CV assay. All agents significantly inhibited both adhesion and biofilm formation in a time- and concentration-dependent way. CSA-131-P was the most effective, preventing adhesion by up to 84% and inhibiting biofilm formation by over 90%. Maximal inhibition of biofilm formation was observed at 1 × MIC, with all ceragenins outperforming meropenem. CSA-131-P and CSA-13 were the most active against mature biofilm. Results also showed that ceragenins inhibited mature biofilm formation more effectively than initial attachment. Overall, CSA-13, CSA-131, and CSA-131-P are promising membrane-targeting candidates for drug development (Damar-Çelik et al., 2021) (**Table 4**).

##### 8.2.2.3 Plant-derived compounds

Several plant-derived compounds have been investigated for their antibiofilm activity against *Achromobacter* species.

Chitosan, a natural polysaccharide obtained from the alkaline deacetylation of chitin, and curcumin, a bioactive compound from *Curcuma longa*, were combined in curcumin-chitosan-sodium tripolyphosphate magnetic nanoparticles via ionotropic gelation. In combination with trimethoprim-sulfamethoxazole, these nanocomplexes inhibited biofilm formation and partially eradicated preformed biofilm of two non-CF *Achromobacter* sp. isolates (Montoya-Hinojosa et al., 2023) (**Table 4**).

Coumarin, a secondary metabolite found in several plant families, has also been explored in the form of coumarin-thiazole hybrids. Among these, compounds 6a and 6c demonstrated moderate antibiofilm effects on one *Achromobacter* sp. isolate, with inhibition levels of 30.2% (± 0.96) and 41.8% (± 2.85), respectively. Mechanistic studies revealed that compounds 6a and 6c induced reactive oxygen species production, disrupted redox homeostasis, and reduced lipid peroxidation, whereas molecular docking and dynamics simulations confirmed stable binding to DNA gyrase, mimicking classical gyrase inhibitors (Ebaid et al., 2025) (**Table 4**).

Plant-derived phenolic acids such as gallic acid and methyl gallate have also been evaluated for their antibiofilm activity on *Achromobacter*. Although gallic acid showed no effect, methyl gallate significantly reduced biofilm formation from five *Achromobacter* sp. isolates (four CF and one non-CF) of respiratory origin, with more than 50% inhibition observed at 1 × MIC, confirming its antibiofilm potential (Flores-Maldonado et al., 2025) (**Table 4**).

Collectively, these studies highlight several natural molecules showing moderate but promising ability to inhibit and/or disrupt *Achromobacter* biofilm.

##### 8.2.2.4 Bacterial-derived compounds

Biosurfactant lipopeptides from *Bacillus subtilis* TR47II, including fengycins and iturins, exhibit antibacterial and antibiofilm activity against one *A. mucicolens* isolated from the buccal cavity of a patient with laryngeal cancer, disrupting mature biofilm by up to 85% in a concentration-dependent way while remaining non-cytotoxic to mammalian cells (De Souza Freitas et al., 2020). Similarly, supernatants from *Lactobacillus plantarum*, which produce various antimicrobial compounds, inhibited biofilm formation of three CF *Achromobacter* sp. but had no effect on preformed biofilm (Danilova et al., 2022) (**Table 4**).

##### 8.2.2.5 Bacteriophages

Phage therapy is emerging as a promising non-antibiotic strategy for *Achromobacter* infections (Li et al., 2026). An ongoing phase 1 randomized trial is evaluating an *Achromobacter*-targeting bacteriophage cocktail in CF patients with chronic lung infection. Although biofilm-related endpoints are not specifically assessed, the intrinsic ability of bacteriophages to penetrate biofilm and infect metabolically inactive bacteria suggests potential efficacy against biofilm-associated infections, which remains to be confirmed *in vivo* (https://clinicaltrials.gov/study/NCT07275905) (ClinicalTrials.gov, n.d.). In addition, a recent study shows that the *in vitro* antibiofilm activity of bacteriophages against *Achromobacter* biofilm-associated infections is enhanced when combined with antibiotics, opening new perspectives for combined antibiofilm strategies (Flores-Ibarra et al., 2026).

##### 8.2.2.6 Miscellaneous compounds

Nanoemulsions are water-in-vegetable-oil formulations produced by dispersing a water-immiscible oil phase into an aqueous phase under high-stress mechanical extrusion. NB-401 nanoemulsion demonstrated bactericidal activity against a single *Achromobacter* sp. isolate; but complete biofilm eradication required it to be used undiluted at 2,000 µg/mL (**Table 4**). Consequently, the potential of NB-401 as a new antimicrobial agent for treating CF biofilm-associated *Achromobacter* infections appears uncertain (LiPuma et al., 2009). Moreover, the general efflux pump inhibitor phenylalanine-arginyl-β-naphthylamide had no effect on *Achromobacter* biofilm formation (Nielsen et al., 2017). Paradoxically, quorum sensing inhibitors, including patulin and penicillic acid, alone or combined with EDTA, increased biofilm formation in two *Achromobacter* sp. isolates from dental unit water lines (Liaqat et al., 2010) (**Table 4**).

Treatment with DNase I reduced biofilm biomass in all three tested *Achromobacter* sp. isolates, whereas subtilisin had strain-dependent effects (a reduction in 2/3 isolates but an increase in one). Both DNase I and subtilisin dispersed 24-h mature biofilm and decreased tolerance to colistin. Alginate lyase reduced biofilm biomass in two isolates, dispersed mature biofilm in only one single strain, and did not affect antibiotic tolerance (**Table 4**). These findings highlight the strain-dependent effects of matrix-degrading enzymes and suggested that targeting several biofilm matrix components may enhance antimicrobial strategies against *Achromobacter* in biofilm-related diseases (Nielsen et al., 2016).

## 9 Concluding remarks

This scoping review highlights biofilm formation as a central, widespread trait of *Achromobacter*, which may contribute to bacterial persistence in both clinical and environmental settings, although further longitudinal studies are needed to support this role. However, it has some limitations. The literature search was conducted in a single database (PubMed-MEDLINE), which may have resulted in some relevant studies indexed elsewhere being missed although screening the reference lists of all included studies led to the inclusion of ten additional articles not selected initially. The substantial heterogeneity in study designs and biofilm assessment methods also limited the availability of sufficiently comparable data and precluded formal meta-analyses. Nevertheless, ordinal biofilm formation data from comparable studies using the CV method and Stepanović’s interpretative criteria were pooled for statistical analyses to compare biofilm formation across *Achromobacter* species and isolate origins. Across the genus, our statistical analyses show that *A. xylosoxidans*, the species most frequently isolated in CF and non-CF infections, exhibits the highest prevalence of biofilm-producing isolates. This enhanced biofilm-forming capacity may provide greater protection against host defenses and antimicrobial exposure (Domouchtsidou et al., 2026) and could therefore represent one of several traits contributing to the epidemiological success of *A. xylosoxidans*, alongside other features previously more frequently reported in this species, like greater antibiotic resistance (Ray et al., 2025; Cultrera et al., 2026), which may facilitate survival under antimicrobial pressure; enhanced swarming motility (Pereira et al., 2017), which could promote access to and colonization of the infectious niche; and higher siderophore production, which may confer an advantage in iron-limited environments (Sorlin et al., 2024). In contrast, biofilm formation did not vary significantly according to the clinical origin of isolates, supporting a strain-dependent rather than CF/non-CF origin-dependent phenotype. This interpretation is reinforced by longitudinal evidence showing various within-host trajectories, as well as by polymicrobial studies illustrating complex reciprocal interactions between *Achromobacter* and other microorganisms.

Among antibiotics demonstrating antibiofilm activity against *Achromobacter* spp., trimethoprim-sulfamethoxazole and cefiderocol appear to be the most effective, based on the evidence gathered from this scoping review, a recent and important finding that should be considered in therapeutic decision-making for the management of *Achromobacter* infections. Several non-antibiotic approaches also show promising experimental potential but require further mechanistic and translational validation prior to clinical application.

Finally, despite growing interest in *Achromobacter* biofilm, its contribution to virulence, clinical outcomes, and treatment failure has been scarcely investigated. However, several studies suggested a possible association between strong biofilm production and increased disease severity (Trancassini et al., 2014; Liu et al., 2016), reported a modest association between biofilm formation and cytotoxicity (Acharya et al., 2026) or described higher carbapenem MICs among strong biofilm producers (Liu et al., 2016), supporting the need for further studies. Future progress will depend on harmonized methodologies, strain-resolved analyses, clinically relevant experimental models that reflect chronic and polymicrobial infection contexts, ultimately supported by well-designed clinical studies. Such approaches will be crucial to guide development of effective antibiofilm strategies targeting these increasingly recognized pathogens.

## Data Availability

The original contributions presented in the study are included in the article and its **Supplementary Table 1**. Further inquiries can be directed to the corresponding author.
